# Photosensitive and pH-dependent activity of pyrazine-functionalized carbazole derivative as promising antifungal and imaging agent

**DOI:** 10.1038/s41598-020-68758-w

**Published:** 2020-07-16

**Authors:** Agnieszka Chylewska, Aleksandra M. Dąbrowska, Sandra Ramotowska, Natalia Maciejewska, Mateusz Olszewski, Maciej Bagiński, Mariusz Makowski

**Affiliations:** 1 Faculty of Chemistry, University of Gdańsk, Wita Stwosza 63, 80-308 Gdańsk, Poland; 2 Faculty of Chemistry, Gdańsk University of Technology, Gabriela Narutowicza 11/12, 80-233 Gdańsk, Poland

**Keywords:** Chemistry, Analytical chemistry, Physical chemistry

## Abstract

Carbazole skeleton plays a significant role as a structural scaffold of many pharmacologically active compounds. Pyrazine-functionalized carbazole derivative was constructed by coupling 2-amino-5-bromo-3-methylaminepyrazine (ABMAP) into 3 and 6 positions of the carbazole ring. Multi-experimental methods were used, e.g.*,* potentiometric, spectroscopic (ATR, UV, XRD powder,^1^H and^13^C NMR), electrochemical (cyclic voltammetry), and optical techniques, to receive the complete structural analysis, physicochemical (pKa, logP) and biological profile of a new carbazole derivative with acronym *3,6-PIRAMICAR*. The interaction ability of the compound studied with potential cellular targets like *Calf Thymus* DNA (*CT*-DNA), or *Bovine Serum Albumin* (BSA) were also taken into account. Experiments showed the existence of strong binding, but no DNA or BSA cleavage was observed*.* The comparative analyzes of compounds anti-*Candida* action clearly show pH-dependent antifungal activity of *3,6-PIRAMICAR*, which was strongly stimulated in the acidic media. Surprisingly, the titled compound turn out to be much more effective (14 times by MIC50; 8 times by MIC; *c.a.* 4 times by MFC) against *Candida krusei* than fluconazole at pH 4.

The emergence of multidrug-resistant microorganisms, as well as fungal infectious diseases, is a major global problem, especially for immuno-deficient populations. The development of new antifungal agents in clinical trials is problematic inferior to the incidence of drug resistance, and the available antifungal agents are restricted. Their mechanisms are based on certain characteristics of the fungus in order to avoid biological similarities with the host^1^. Fungi in the *Candida* genus are the most common fungal pathogens which can colonize various host niches (stomach, vagina and oral mucosa) with varying ambient pH range^2–6^.

Carbazoles and derivatives with carbazole skeleton are currently tested intensively according to their antimicrobial properties which is directly relating with their structure or in more details with anaromatic N-heterocyclic ring inside^7^. Carbazole derivatives have been reported to be potential agents against tumor^8^ or opportunistic infections of AIDS^9,10^. The studies about high active DNA intercalators show the significance of the carbazole structural agents like planarity or aromaticity^11,12^. Interestingly, the mentioned parameters are described as responsible for the high affinity of carbazoles substituted at 3,6- or 2,7-positions by amine, amidine, or imidazoline groups to GC or AT-rich sequences of DNA^13,14^. However, chemistry of especially di-substituted 3,6-derivatives of carbazole is still not very extensively explored due to problems with synthesis of such compounds. Mainly nitrogen atom was a hot spot for carbazole derivatives^14^.

The first concept of the studies was to check the basic design structures assumptions to predict pharmaceuticals activity, like presented carbazole functionalized by pyrazines—our previous research objects. Consequently, the confirmation of the possibility of one direction antimicrobial properties transfers, from substituents to the main carbazole motif, was initially considered as a general project objective. The *3,6-PIRAMICAR* properties of mimicking the pyrazinamide antibacterial action were also taken into account because it is typical to test at the same time antibacterial and antifungal activity. Interestingly, the proved lack of *3,6-PIRAMICAR* antibacterial activity forced us to generate and applicate of a new research approach. Firstly, the pathogens were changed in tests based on the predicted similarity of unknown *3,6-PIRAMICAR* to pyrazine derivatives antifungal action (selectivity against *Candida albicans*) studied in our previous research^15–18^. Additionally, we applied the pH conditions analogy (values lower than 5.5) for the pyrazinamide activation. Note, the synthesized *3,6-PIRAMICAR* is the article object explored as novel alternative to fluconazole treatments. Antifungal activity and lack of antibacterial activity, on the other hand, can be an advantage because such compounds are more selective.

The pH profile of 3,6-di-(2-amino-3-(methylamino)pyrazino)-9H-carbazole (*3,6-PIRAMICAR*), see Fig. 1, and distribution diagrams of its species would provide the knowledge about the extent to establish the proper ionization state of compound (also considered as potent drug or pro-drug) presents at physiological pH. Electrochemistry of carbazole compounds is an object of interest mainly because of their applications^19–23^. However, it has been shown that the mechanism of biological action of some biologically active compounds is associated with their redox activity^24,25^. The aforementioned comparisons were expected to lead to solving research problems. The first one is related to design and determination of optimal conditions for conducting synthetic pathways to receive the *3,6-PIRAMICAR*. The second is to receive the data confirming or refuting the thesis about high biological potential and activity of *3,6-PIRAMICAR* as a representative of a class of symmetrical carbazole derivatives. Moreover, biological evaluation of the compound was assessed by in vitro assays to explore anticancer and antimicrobial activity. The cytotoxic effect was determined for two cancer cell lines (i.e., non-small cell lung cancer—A549 and human colorectal cancer—HCT116). The antifungal activity was determined using *Candida albicans*, *Candida glabrata,* and *Candida krusei* strains of fungi at various ambient pH values.

**Figure 1:**
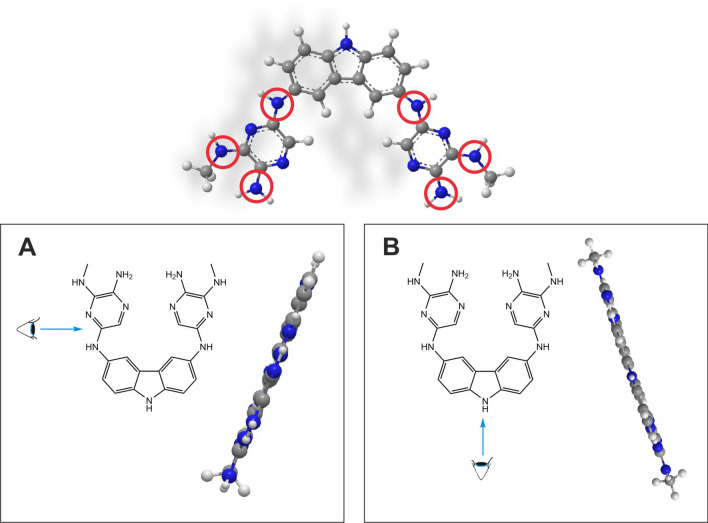
*3,6-PIRAMICAR* structure: **A** one side view **B** central view.

Herein, we report in detail and for the first time to the best of our knowledge, the synthesis, physicochemical properties, biological activity, and application potential of *3,6-PIRAMICAR*.

## Results and discussion

### Proton dissociation processes

**Figure 2:**
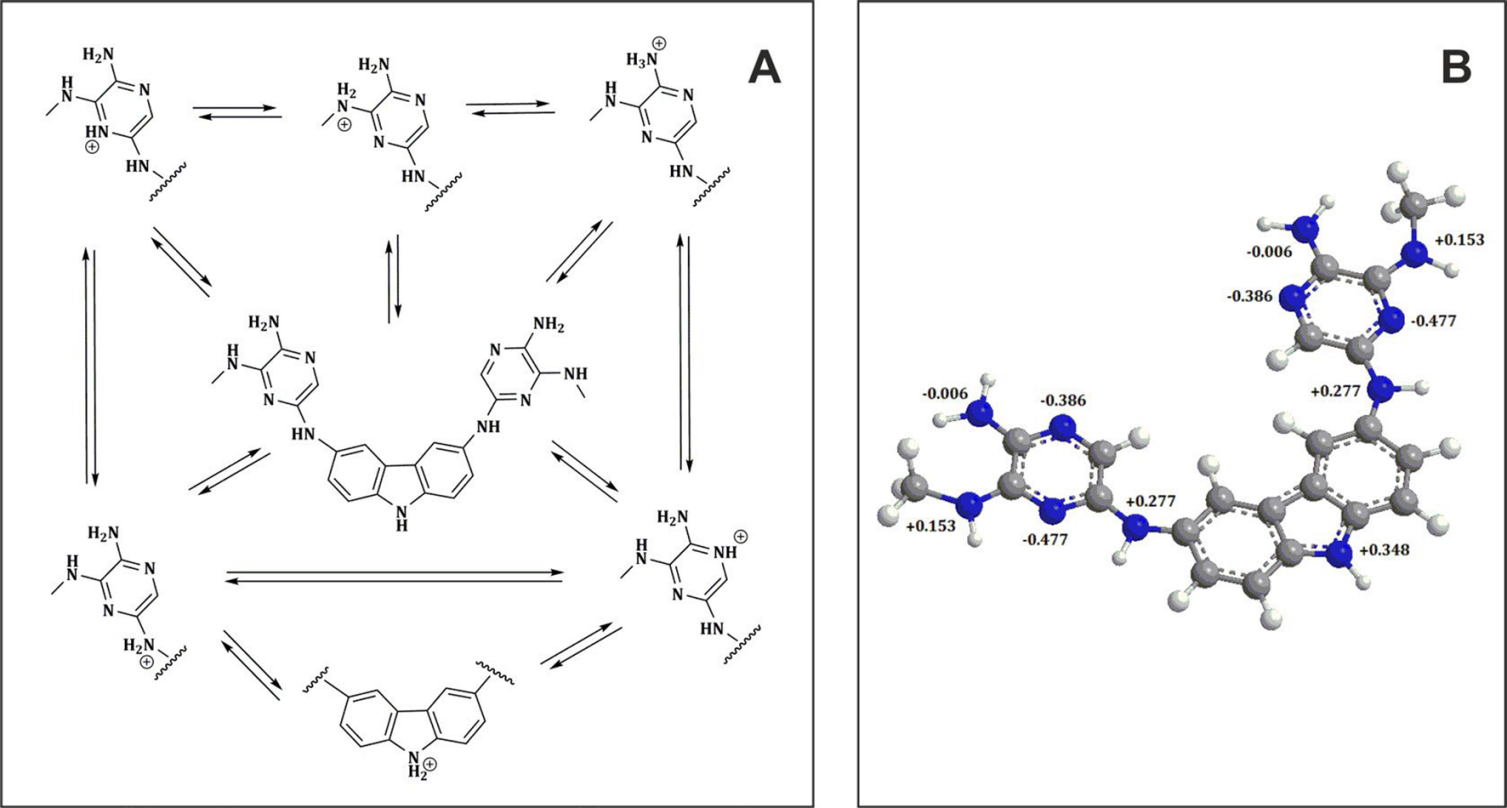
Presumably acid–base equilibria of *3,6-PIRAMICAR* in different pH aqueous solution with suggested microscopic forms; **B** charge distribution on nitrogen atoms in *3,6-PIRAMICAR (*generated with *ChemBio3D Ultra* programme).

A neutral (unionized) molecule of *3,6-PIRAMICAR* contains potentially six (or eleven, together with N-heterocyclic atoms) basic sites (Fig. 1). The knowledge about the potential protonation places (atoms) in structure as well as ionization state of compound active (neutral or ionized) in solution at different pH is crucial. The scientific reports prove that pyrrole protonation overlaps on an α-carbon atom^26^. In the case of indole derivatives—β-carbon^27^ is preferred place to accept the proton. The mentioned proofs suggest that for higher analog of pyrrole (indole) ring—carbazole, second side of the pyrrole ring should be blocked and as the first, the N atom of indole should be protonated. Moreover, the protonation process of *N*-heteroaromatic atoms in derivatives typically described in the literature^28^ occurs in very strong acids like concentrated (70%) perchlorate acid. The protonation reaction of *3,6-PIRAMICAR* was prepared in the mentioned conditions and registered spectrophotometrically as evidenced by the reversible change presented in Supplementary Information Fig. S1A online. The principal bands in the UV spectrum proved that the protonation process occurs on N atoms. Protonation points correlate very well with Hammett constants (H_0_), which were, therefore, used to estimate basicity (see Supplementary Fig. S1B online). This provides an upper limit for the basic strength of the *N*-heteroaromatic atoms of a studied compound, in which equilibrium protonation occurs preferentially on carbon and for which nitrogen basicity is not directly measurable^29^. To obtain the pKa values of *N*-heteroaromatic atoms, the Hammett acidity function was implied to the UV-spectrophotometric titrations of *3,6-PIRAMICAR* samples by using 70% HClO_4_ as a titrant. The values of pKa_1_, pKa_2_, and pKa_3_ constants, respectively, were calculated based on modified Eq. (2), and their values were summarized in Table 1. It can be seen (Fig. S1B) that the correlation between experimental and fitting data line obtained from calculations at 232 nm are in a reasonably good agreement. The hybrid potentio-spectrophotometric titration method was used to determine the protonation constants of *N*-aliphatic atoms. The recorded pH-dependent spectra (Fig. S2 in *SI*) revealed some of the proton dissociation processes at wide range pH; the calculated protonation pKa constant values are reported in Table 1. Titrations showed characteristic spectral changes in the wavelength range of 200–450 nm. *3,6-PIRAMICAR* displays intense absorption bands in all the protonation forms at 245 nm, 292 nm, 314 nm, and 331 nm, which are assigned to n → π* transitions. The determined pKa values demonstrate that the proton dissociation processes overlap, and the neutral form predominates in the physiological pH range, as illustrated in Supplementary Fig. S3B. Figure 2A shows all possible protonation sites for all nitrogen acceptor atoms occurring in the *3,6-PIRAMICAR* molecule. The value of pK_1_ can most presumably be attributed to the deprotonation of the 2°amine unit (inside of two heteroaromatic rings), while pK_2_ belongs to the deprotonation of the 2°amine unit (between the aliphatic substituent and heteroaromatic ring). Our assumptions are based on the charge distribution map determined using the Hückel method, as shown in Fig. 2B.

**Table 1:** The pKa values of *3,6-PIRAMICAR* obtained by two different methods. All experimental research were recorded at 25.0 ± 0.1 °C.

Method type	pH range: (*H*_*0*_ range)	pKa values		
		pKa_1_ ± SD	pKa_2_ ± SD	pKa_3_ ± SD
Potentiometry titration	(3.05÷10.90)	3.67 ± 0.20	6.39 ± 0.08	10.86 ± 0.47
UV titration	(3.05÷10.90)	2.92 ± 0.17	6.27 ± 0.07	9.58 ± 0.08
UV Hammett titration	(+ 1.00÷− 4.50)	− 4.49 ± 0.07	− 3.35 ± 0.09	0.10 ± 0.09

*3,6-PIRAMICAR* contains two acidic amide groups (–NH–) and one basic amine group (–NH_2_), which correspond to p*K*a_1_, p*K*a_2,_ and p*K*a_3_ respectively. The amine group can attach a proton, while the amide group is able to detach a proton at specific pH conditions. The pKa of the *3,6-PIRAMICAR* was determined by potentiometric titration, and the titration curve was shown in Fig. S3. Results indicate that the pKa determined by potentiometric titration agrees with most spectroscopic data. The titration curves obtained for *3,6-PIRAMICAR* (Fig. S3A) show three potential changes. In the presented figure, there is a comparison of experimental data (black squares) and data obtained from the calculation by using CVEQUID program (solid line). It can be seen that the correlation between these two results is, in fact, very good for each compound studied.

### Redox profile

The influence of the environment on the electrochemical characteristics of compounds included carbazole skeleton turned out to be extremely interesting. Carbazole compounds oxidize, forming the cation radicals. Their strongly π-excessing rings tend to undergo polymerization after oxidation^30,31^. As the carbazole cation radical is very unstable, it can couple with either another cation radical or with a parent molecule, forming the more stable di-carbazyl (dimer). The oxidation of carbazole is rather complex and has the characteristics of an electrochemical-chemical-electrochemical (ECE) process in which one electron is involved per parent molecule. Interestingly, the carbazole amine derivatives show a slightly different nature of oxidation processes^32^. The presence of additional nitrogen atoms supplying electrons in the molecule reduces the initial oxidation potential compared to carbazole^33^. 3,6-di-amino-9H-carbazole, used as a substrate for the synthesis of *3,6-PIRAMICAR*, in an aprotic solvent, showed a small oxidation signal at the potential of Ea_1_ = 0.480 V and an intense anode peak at the potential value of Ea_2_ = 1.530 V (Fig. S4A in SI). The second peak of the reduction process appeared only when registering the first scan. During the next cycles, the first reduction peak also disappears. Reregistering of both signals in their initial form is only possible after cleaning the working glassy carbon (GC) electrode. The voltammogram registered for the second substrate used for the synthesis of *3,6-PIRAMICAR*, i.e.*,* 2-amino-5-bromo-3-(methylamino)pyrazine (ABMAP), showed two irreversible oxidation processes (Fig. S4B in SI). The values of anode potentials were 0.925 V and 1.408 V for Ea_1_ and Ea_2_, respectively. These peaks did not change their positions or intensities during the recording process of subsequent measurement scans.

In the aprotic solvent, two oxidation peaks were observed for the *3,6-PIRAMICAR* compound (Fig. S5). The impact of scanning rate, in the range from 25 to 500 mV s^−1^, on the intensity and location of redox processes was investigated. The increase in the speed of recording redox processes seemingly caused an increase in the intensity of signals and shifted them towards more positive potential values. However, due to high background currents occurring at high scanning rates, the value of 100 mV s^−1^ was chosen for measurements. The values of oxidation potential at this scan rate were of Ea_1_ = 0.910 V and Ea_2_ = 1.483 V. These values are very similar to the value of the potentials of anode processes registered for 2-amino-5-bromo-3-(methylamino)pyrazine.

**Figure 3:**
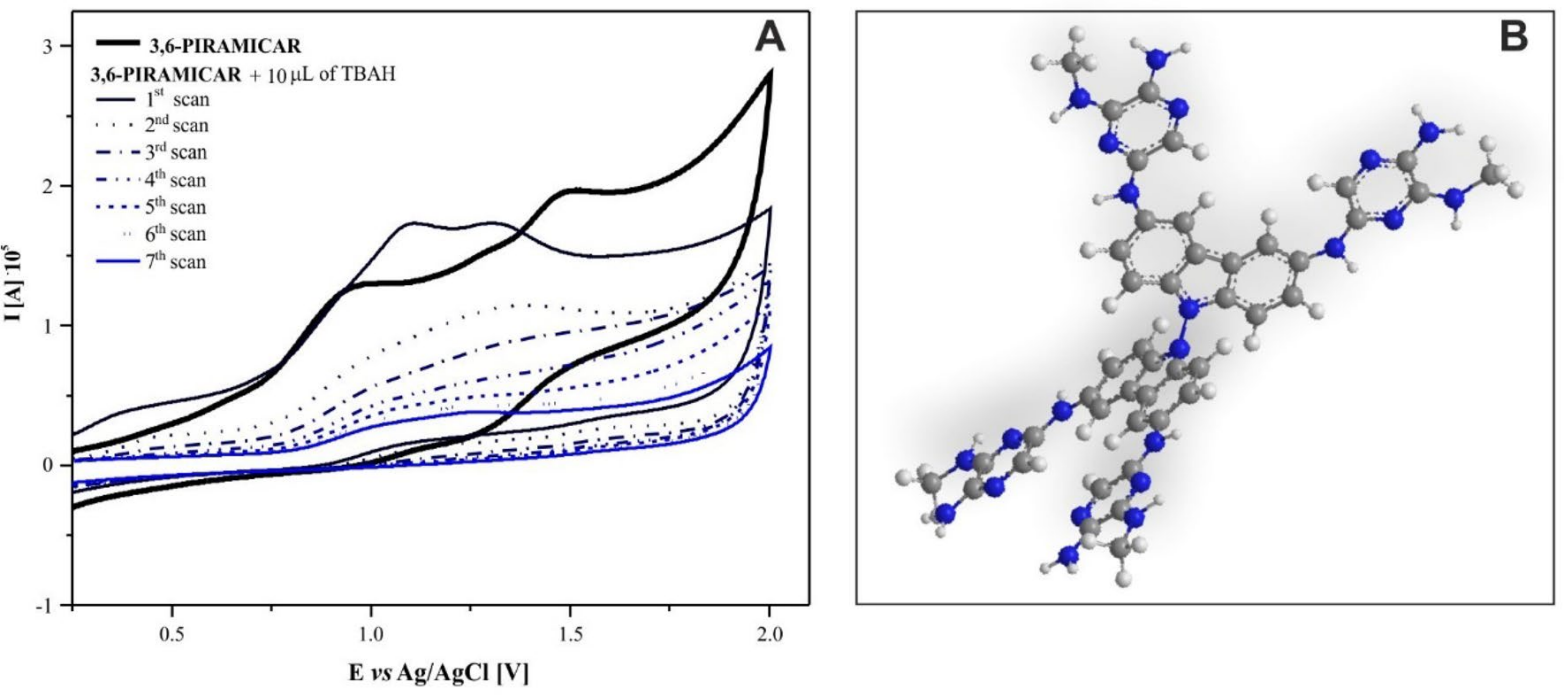
**A** *3,6-PIRAMICAR* voltammograms after addition of tetrabutylammonium hydroxide (TBAH) solution (100 mV/s; in acetonitrile)—time scanning (every 2-min scan); **B** Proposal structure of *3,6-PIRAMICAR* dimer formed as a result of electrochemical process in basic medium.

Moreover, the peaks generally maintain their intensity during the recording process of subsequent voltammograms. These factors indicate that the group responsible for signaling redox processes in the *3,6-PIRAMICAR* molecule are mainly pyrazine substituents, not the carbazole moiety itself. Our results have shown that the environment (pH of the solution) has a strong impact on the redox activity of the compound. The addition of subsequent portions of 0.1 M tetrabutylammonium hydroxide (TBAH) solution (Fig. S6A) first caused the oxidation peaks to overlap each other, and then reduce signals intensity. In turn, the electrode process in the presence of 0.1 M methanesulfonic acid (MSA) causes precipitation formation in the measurement cell (Fig. S6B). Interestingly, even after the addition of one portion (10 µL) of TBAH, in consecutive measurements (without changing conditions), gradual changes were observed (Fig. 3A). The voltammogram recorded immediately after adding the base and mixing the solution by passing argon, showed two oxidation peaks approaching to each other. On the following recorded scans, a gradual disappearance of these signals was observed. Specific reduction of signal intensity in subsequent scans was also observed in the case of 3,6-di-amino-9H-carbazole (Fig. S4 in SI). This may suggest an increasing role of the aminecarbazole moiety in undergoing redox processes. For unsubstituted carbazoles, the 3, 6, and 9 positions are readily available as coupling sites. Substitution of the molecule in 3 and 6 positions eliminates their participation in the dimerization process, and thus the only likely coupling sites remain the 9(N) and optionally positions 1 or 8, respectively^30–32^. The type of dimer formed during oxidation depends on proton concentration in the area around the electrode, the status of the carbazole nitrogen (protonated or unprotonated), and the electron densities at the remaining reactive coupling sites^33,34^. The addition of tetrabutylammonium hydroxide solution increases the possibility of dimerization at 9-position of carbazole (Fig. 3B), by reducing the concentration of protons. The changes observed in the alkaline environment demonstrated the influence of both pH and kinetics on the oxidation process. Subsequent reactions of both cation radicals and dimers formed during the oxidation process, leading to polymerizationand the formation of more complex products that do not show oxidation signals seem to be possible^35^.

### Interactions with biomolecules

**Figure 4:**
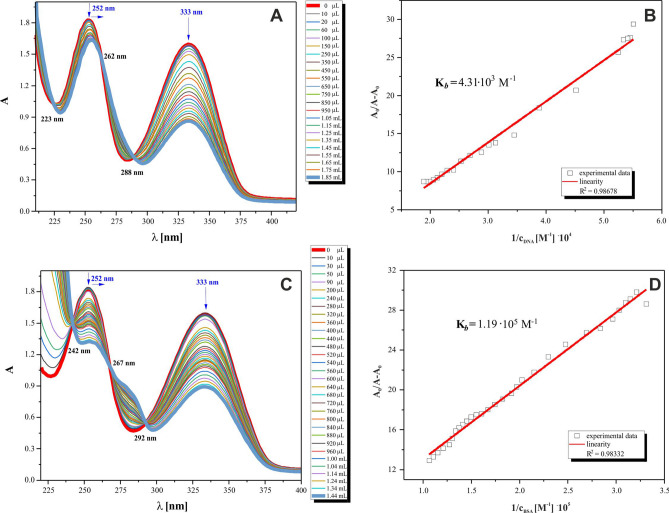
Absorption spectra of *3,6-PIRAMICAR* (0.24 mM) in Tris–HCl buffer upon addition of **A** *CT*-DNA (0–53 μM) with subtraction of the *CT*-DNA absorbance, **B** Plot of A_o_/(A − A_o_) versus 1/[DNA] of *3,6-PIRAMICAR*, **C** BSA (0–94 μM) with subtraction of the BSA absorbance. **D** Plot of A_o_/(A-A_o_) versus 1/[BSA] of *3,6-PIRAMICAR.* The arrows show the absorbance changes related with increasing biomolecules concentration.

The binding affinity of *3,6-PIRAMICAR* to *Calf Thymus*-DNA(*CT*-DNA) as well as to *Bovine Serum Albumin* (BSA) was studied by electronic absorption microtitrations with biomolecules treated as titrants. The representative UV spectra of isolated *3,6-PIRAMICAR* and its adducts with *CT*-DNA and BSA are given in Fig. 4. The complete number of titration spectra obtained together with the initial spectra of biomolecule titrants was collected in Supplementary Figs S7–S12 online.

The increasing DNA, as well as BSA concentrations, led to the substantial decrease in the absorption of *3,6-PIRAMICAR* with slight bathochromic effects at wavelength region about 250 nm for both experiment types. The intercalation mode of compound interactions with DNA is usually presented in the literature by hypochromic and bathochromic effects^34^ observed during electronic absorption titrations. The absorption band intensity for *3,6-PIRAMICAR* at 252 nm (7,740 dm^3^ mol^−1^ cm^−1^) and 333 nm (6,728 dm^3^ mol^−1^ cm^−1^) was decreasing with increasing of DNA concentrations. Based on the above, we observed the mentioned phenomena about hypochromic and redshift effects between 250 and 290 nm (Fig. 4A, C) in the presence of DNA or BSA interacted with *3,6-PIRAMICAR*. The results obtained for *3,6-PIRAMICAR*-DNA system studied, together with the knowledge about 3,6-disubstituted carbazole derivatives as compounds more specific for the GC sequence (than for AT)^13^, allow to suggest that the intercalation mode is quantitatively more competitive with groove binding.

It is worth noting that the intrinsic binding constants (K_*b*_) of *3,6-PIRAMICAR* with DNA and with BSA were determined from the decay of the absorbance monitored for compound studied. These parameters K_*b*_ were evaluated from the Benesi-Hildebrand Eq. ^36^. The K_*b*_ value is related to change in absorbance [A_o_/(A − A_o_)] for pure compound studied (A_o_); for its adduct with a biomolecule (A) versus c^−1^_BIOMOLECULE_. The higher value of binding constant obtained for *3,6-PIRAMICAR*-BSA system (1.19·10^5^ M^−1^) proves the higher *3,6-PIRAMICAR* affinity to association/bind with albumin than with *CT*-DNA (4.3·10^3^ M^−1^). Moreover, the presence of three isosbestic points for both titration types experiments can be observed (Fig. 4), which indicates the strong interactions between an aromatic *3,6-PIRAMICAR* chromophore and each biomolecule studied.

### Biological evaluation

Fungal infections occur all over the world in parallel with the increasing number of people with immunosuppressed. Fungi in the *Candida genus* are the most common fungal pathogens which can colonize various host niches (stomach, vagina, and oral mucosa) with varying ambient pH range^1^. Drug molecules with the carbazole framework possess a wide range of biological and pharmacological activities^37–40^. Thus, the antiproliferative activity of *3,6-PIRAMICAR* and its substrates was evaluated against human non-small cell lung cancer (A549) and colorectal carcinoma cell line (HCT116) for 72 h, using MTT assay. Investigated compounds, tested in the 250–0.4 μM concentration range on both tumor cell lines showed no anticancer activity (Table S1 in SI). Next, antimicrobial potency towards fungal species was investigated by the broth serial dilution technique. Table 2 outlines MIC50 and MIC susceptibility results, including MFC of compounds, tested at pH values of 4, 5.5, and 7, respectively, for all tested yeast strains.

**Table 2:** Antifungal activities of compounds against Yeasts (MIC, MIC50, MFC μM).

Strain	pH	3,6NH_2_			*3,6-PIRAMICAR*			Fluconazole		
		MIC50	MIC	MFC	MIC50	MIC	MFC	MIC50	MIC	MFC
*C. albicans*	7.0	> 250	> 250	> 250	> 250	> 250	> 250	7.46 ± 3.69	125	250
	5.5	98.26 ± 22.52	> 250	> 250	35.26 ± 9.26	250	250	7.71 ± 2.81	> 250	> 250
	4.0	113.25 ± 12.95	> 250	> 250	106 ± 13.78	250	> 250	5.89 ± 2.09	> 250	> 250
*C. glabrata*	7.0	> 250	> 250	> 250	> 250	> 250	> 250	19.99 ± 5.45	62.5	125
	5.5	> 250	> 250	> 250	> 250	> 250	> 250	229.6 ± 28.96	> 250	> 250
	4.0	63.22 ± 8.14	125	250	30.88 ± 6.56	62.5	125	> 250	> 250	> 250
*C. krusei*	7.0	> 250	250	> 250	> 250	> 250	> 250	8.15 ± 3.13	62.5	125
	5.5	82.12 ± 9.24	> 250	> 250	78.11 ± 8.24	> 250	> 250	8.75 ± 4.65	250	> 250
	4.0	31.94 ± 7.86	125	250	10.05 ± 3.54	31.25	62.5	140.01 ± 26.05	250	> 250

**Figure 5:**
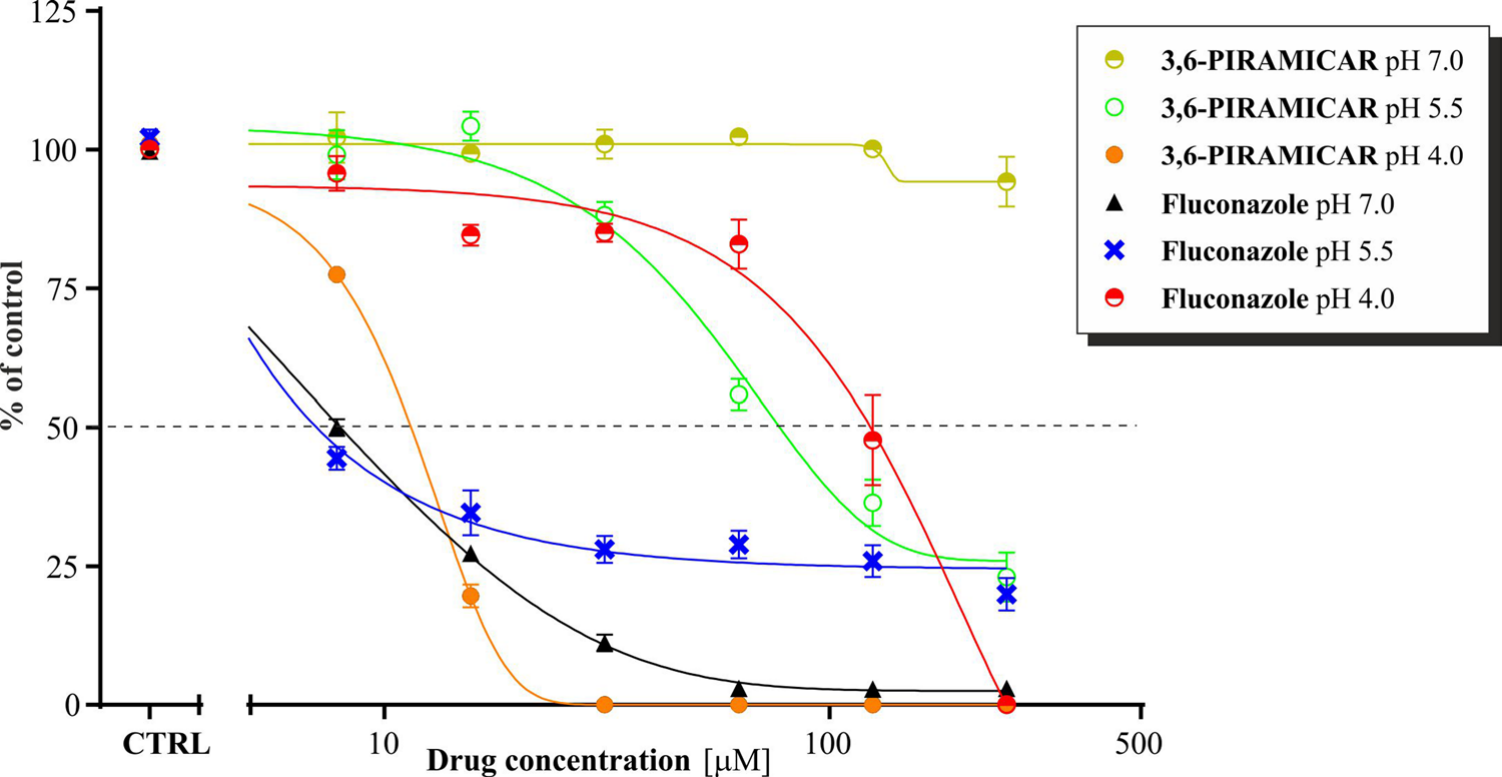
Comparison of *3,6-PIRAMICAR* and Fluconazole antifungal activity against *Candida krusei* at pH 7.0, 5.5 and 4.0.

Irrespective of pH, the *3,6-PIRAMICAR* compound is more active than its substrates. At pH 7, none of the isolates was susceptible to investigated compounds. In general, with a progressive reduction at pH after 24 h of treatment, a significant decrease in MIC was evident with *3,6-PIRAMICAR* and with 3,6-NH_2_, in comparison to fluconazole whose activity decreased with a descent at pH (Fig. 5, Figs S13–S15 in SI)^41^.

ABMAP was not active against any of the tested strains. The MIC50s of *3,6-PIRAMICAR* for *Candida glabrata* and *Candida krusei* were 30.88, 10.05 μM, respectively, while the MICs values for these strains were 62.5 and 31.25 μM, respectively. *Candida albicans* was the most susceptible to *3,6-PIRAMICAR* at pH 5.5 with MIC50 35.36 μM, while MIC was 250 μM. The highest antifungal activity of *3,6-PIRAMICAR* was shown against *C. krusei* at pH 4, followed by MFC 62.5 μM. Compounds were also tested on fluconazole-resistant clinical isolates of different *Candida* species (Table S2 and S3. in SI). Out of the all tested structures, *3,6-PIRAMICAR* was the most active at pH 4 toward *C. krusei* (74) with MIC50 98.97 μM*.*

### Photosensitive nature

**Figure 6:**
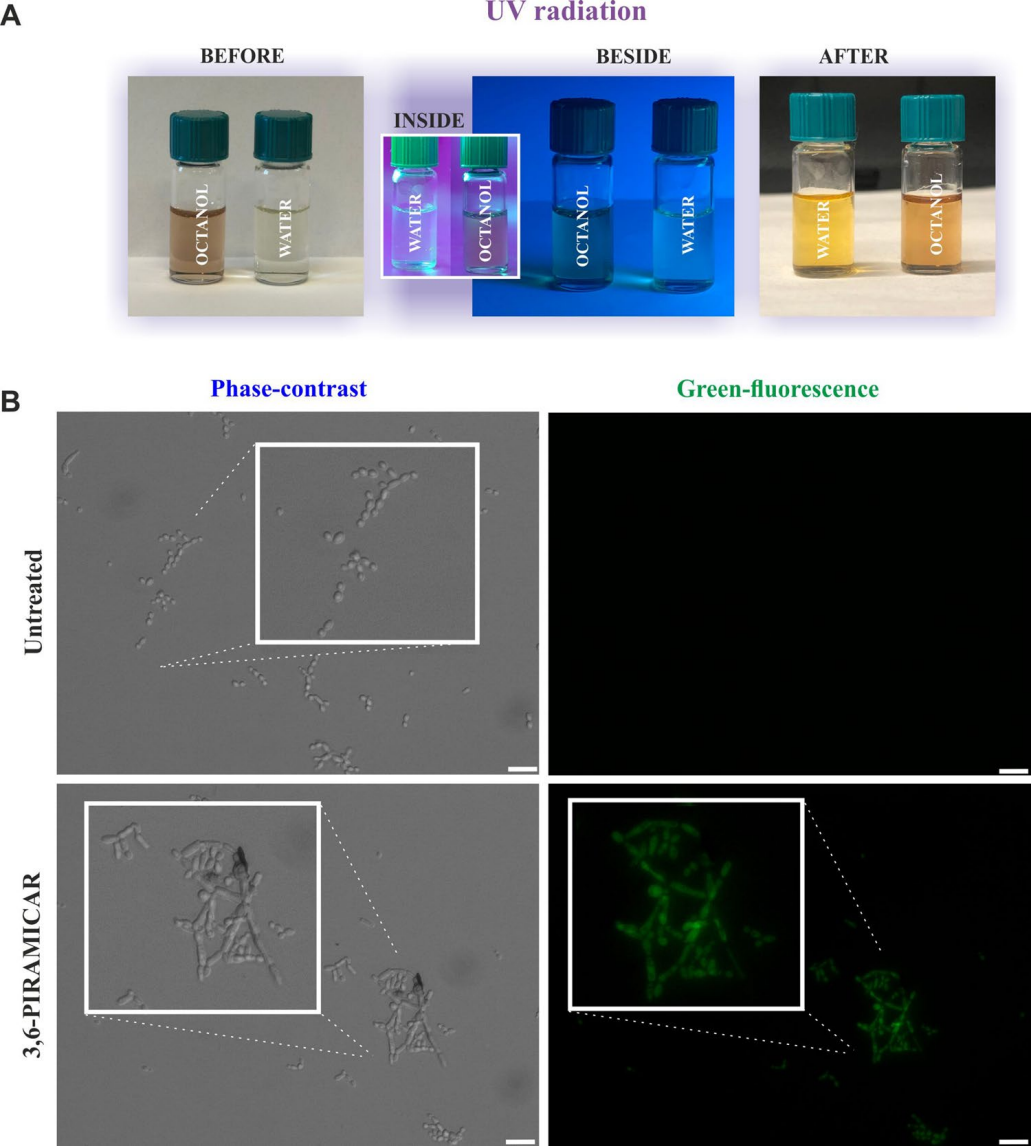
**A** Images of the experimental effects related to the *3,6-PIRAMICAR* behavior exposed to UV light. **B** *Candida krusei* cultures in broth medium after exposure to *3,6-PIRAMICAR* (scale bar = 20 μm) analyzed by fluorescent microscopy method.

**Figure 7:**
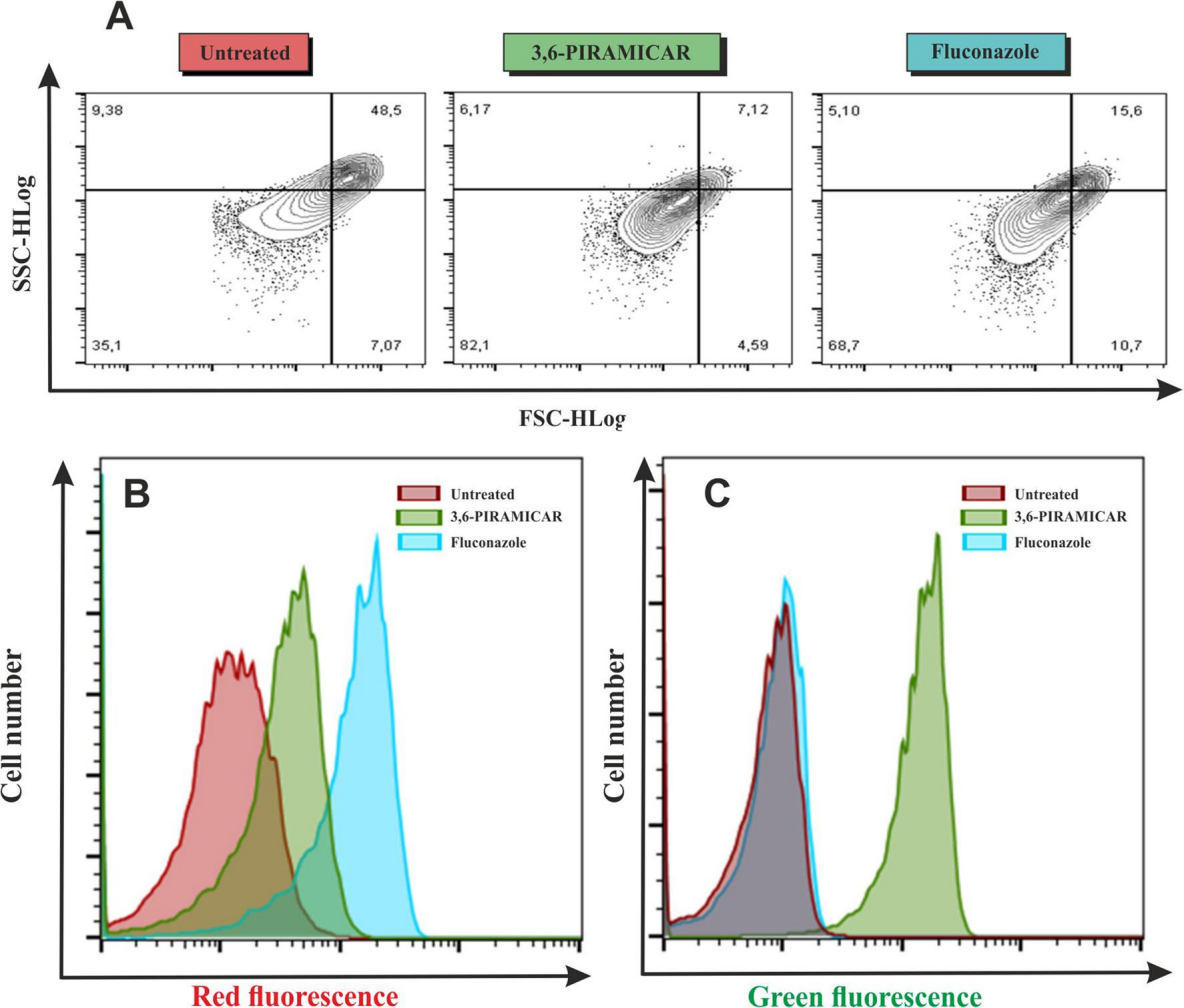
Flow cytometric analysis: **A** Representative dot-plot scatter analysis of *Candida krusei*; **B** Analysis of fungi labeled with propidium iodide for detection membrane permeability in *3,6-PIRAMICAR* and fluconazole treated *Candida krusei*; **C** Analysis of accumulation of *3,6-PIRAMICAR* in *C. krusei* after 24 h of treatment.

The hydro- or lipophilic character and ionization ability are the physicochemical properties used to predicts the general pharmaceutical form dominating at particular conditions. The experimental data, as well as detailed calculation parameters related to log(P)/(D) of *3,6-PIRAMICAR* obtained as a result of the spectroscopic investigations, were presented in graphics and collected summarized in Supplementary Information Figs S16–S20 online. The *3,6-PIRAMICAR* partition (distribution) coefficient results were also presented in Table S4 of SI. They confirmed the *3,6-PIRAMICAR* properties with strong similarity to those of known lipophilic drugs (value of logP above 1.1) characterized by the high value of logP like metoprolol (selective bet-1 receptor blocker used in the treatment of cardiovascular system diseases; logP 1.87), bisoprolol (selective beta-1 adrenergic receptor antagonist with antihypertensive activity; logP 1.87) or amphetamine (poison, synthetic drug; logP 1.85)^42^. Interestingly, the photosensitive properties of *3,6-PIRAMICAR* were revealed in our measurements. Its aqueous and octanol solutions freshly prepared exhibited the fluorescent glows, which were visible, especially in the case of the dilution process. Moreover, the radical change of colors for both *3,6-PIRAMICAR* solutions exposed to UV light was observed (Fig. 6A). This observed phenomenon resembles the behavior of known fluorescent dyes like fluorescein and eosin used in the imaging process. Based on the above, it could be assumed that the compound tested might have been applied, for example, in microscopy as a marker in cells, nucleic acids, proteins, antibodies, etc*.* or in geology and also in environmental protection (to track watercourses and leaks from pipelines). Fluorescence microscopy (Fig. S15 in SI; Fig. 6B) and flow cytometry (Fig. 7) revealed that *3,6-PIRAMICAR* accumulate after 24 h of incubation in *Candida krusei* cells cultured in broth RPMI 1640 or solid YPD medium buffered to pH 4 as evidenced by green fluorescence.

## Flow cytometric assay

Morphological changes of *C. krusei* cells induced by *3,6-PIRAMICAR* were observed by flow cytometric dot plot analysis (Fig. 7B, C) by comparing cell profiles of the FSC as cell size indicator and SSC as granularity indicator. There was a significant increase of cell number with lower FCS values relative to the population of untreated cells (35.1 ± 3.2%)in cells exposed to *3,6-PIRAMICAR* (82.1 ± 3.1%) and fluconazole (68.7 ± 2.1%). Shrinkage of the cells is occurred by increased membrane permeabilization by pore, leading to loss of cytosolic ions or intracellular components resulting in cell cycle arrest^43^.

To elucidate the mode of antifungal action of compounds treatment, we investigated the effect of *3,6-PIRAMICAR* and fluconazole on the membrane's structure of *C. krusei* using propidium iodide influx assay (Fig. 7B). Propidium iodide (PI) is a DNA intercalating fluorochrome used for viable cell exclusion that only penetrates cells with the disruptive plasma membrane^44^.In cells exposed to treatment at pH 4, increased fluorescent intensity (31.1 ± 5.3%) by flow cytometry was observed with 62.5 μM *3,6-PIRAMICAR* compared to untreated cells (1.9 ± 4.2%). However, this effect was lower than with fluconazole (81.4 ± 6.12%). These data indicate that *3,6-PIRAMICAR* reduces the integrity of cell membranes at acidic pH values. Based on that, it can be proposed that *3,6-PIRAMICAR* interacts with fungal cells membranes via electrostatic interaction because of its cationic charge what results in membranes cell damage. The loss of cell membrane integrity due to the action of *3,6-PIRAMICAR* could cause plasma membrane depolarization and cell death.

## Methods

The idea of designing the symmetrically substituted carbazole as a central pharmacophore segment tethered with one-type pyrazine was related to stimulation of with desired pharmacological behavior. The lipophilicity control in the proposed scaffold could be accomplished with the choice of appended N-heteroaromatic pyrazine. The common approach to achieve the symmetrically substituted carbazole allowed to obtain the product **(4)** with acronym *3,6-PIRAMICAR*. Herein, we report an efficient four-steps one component synthesis of novel carbazole motif 3,6-disubstituted by pyrazine. To generalize the reaction, the 3,6-diiodo-9H-carbazole was blocked by tosyl chloride (TsCl) on N-carbazole atom in the initial step to allow reactions with pyrazine derivatives. All the reactions proceeded reasonably well and the products **(1)–(4)** obtained were rigorously purified (> 95%) over silica gel column chromatography. Pyrazines functionalized carbazole were fully characterized by their^1^H NMR, ATR and mass (EI-MS) spectral data.

The main assumption, reactions and precursors of the synthetic pathway were presented together in Fig. 8. The experimental details together with structural analyses as well as the adequate procedure chemical and biological conditions were clearly explained and summarized in Supporting Information file (SI), Figs. S21–S29.

**Figure 8:**
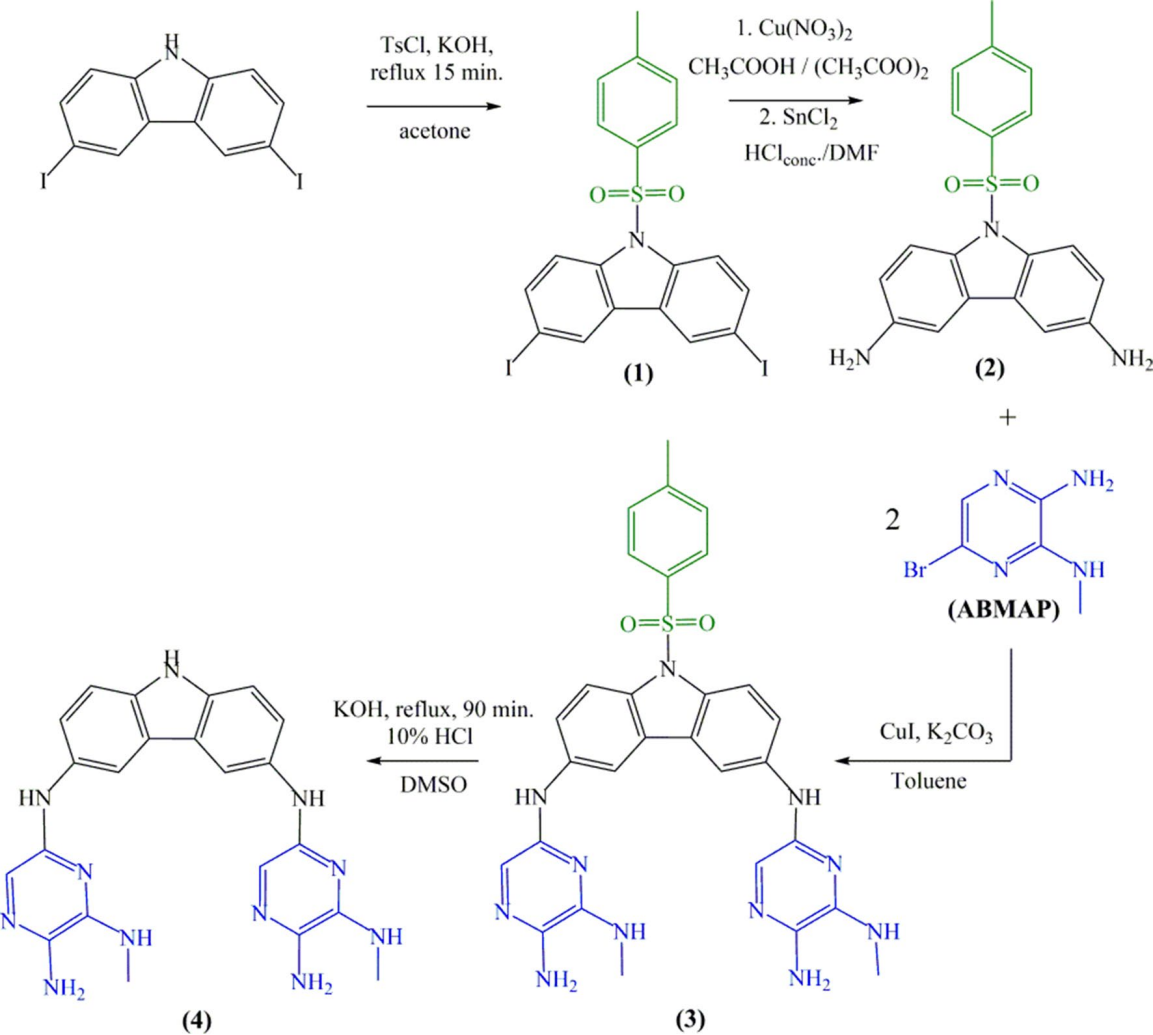
Synthesis pathway of the *3,6-PIRAMICAR.*

### Chemical evaluation

The structural analyses of *3,6-PIRAMICAR* synthesized were prepared by using four independent analytical techniques. The first one, elemental analysis leads to obtain the percentage compositions of the elements by an element analyzer Carlo Erba EA 1108 CHNS. The second, MS leads to receive the mass spectrum (Fig. S21 in SI) of the compound studied (positive polarization) which was registered on MALDI-TOF Biflex III apparatus (Brücker). The third one, ATR spectroscopy makes possible to record the oscillatory spectra of *3,6-PIRAMICAR* in the wave number range 4,000–450 cm^-1^ on a Spectrum Two FT-IR instrument (Perkin Elmer). Here, the solid states of *3,6-PIRAMICAR* compound studied together with its precursors, and additional reference samples like 9H-carbazole, 3,6-di-amine-9H-carbazole and 2-amino-5-bromo-3-(methylamino)pyrazine (ABMAP) were used to analyze similarities and differences in signals observed between substances selected. Finally,^1^H,^13^C NMR, XRD powder spectra of *3,6-PIRAMICAR* were collected in *Supporting Information* file (see Supplementary Figs S22–S28 online*,* respectively). Both NMR spectra were obtained by AVANCE 400 MHz and D2 PHASIER spectrometers (Brüker) at the Faculty of Chemistry (University of Gdańsk). Additionally, the biomolecules (DNA and BSA) intermolecular interactions studies were monitored by electronic spectra recorded on an Evolution 300 spectrophotometer in the range of 200–450 nm (spectral bandwidth 2 nm; double beam mode with zero-baseline correction). Furthermore, two spectroscopic experiments were carried out for *3,6-PIRAMICAR* partition coefficient determination, which experimental conditions, as well as their graphical forms of results, were included in the *Supporting Information*. We used a jacketed titration cell connected to a constant temperature water bath set to 25.0 ± 0.1 °C in all spectroscopic measurements performed.

The electrochemical measurements were carried out in a single-compartment, three-electrode cell with an Autolabpotentiostat/galvanostat PGSTAT204 (MetrohmAutolab B.V., The Netherlands) controlled by the Nova software. For the measurement of electrochemical characteristics, a concentrated solution of *3,6-PIRAMICAR* with approximately 0.5 mM was used. Concentrations of substrate solutions (3,6-di-amine-9H-carbazole and 2-amino-5-bromo-3-methylaminopyrazine) were about 1 mM, respectively. The analyzed compounds were dissolved in acetonitrile together with the 0.1 M tetrabutylammonium perchlorate (TBAP). The working electrode was glassy carbon electrode of 2 mm diameter, carefully polished before each experiment using 0.5 μm alumina suspension (Buehler). The values of potential were measured versus a double junction silver/silver chloride (Ag/AgCl) reference electrode with the aqueous sodium chloride (1 M NaCl) solution in 0.1 M TBAP in methanol. The platinum wire served as an auxiliary electrode. Solutions of metanesulfonic acid and tetrabutylammonium hydroxide were used to determine the effect of pH on electrochemical characteristics. All solutions prepared were degassed by passing argon. All measurements were performed at least two times independently at 25.0 (± 0.1 °C).

The procedure of potentiometric and spectrophotometric detailed description was included in *SI*.

#### Affinity to biomolecules assay

UV–Vis titration assay was carried out in the tris-(hydroxymethyl)-amino methane (Tris–HCl) buffer solution (5 mM Tris–HCl, 50 mM NaCl, pH 7.39). A solution of *CT*-DNA (Sigma-Aldrich)in Tris–HCl gave a ratio of UV–Vis absorbance of 1.8–1.9 at 258 and 280 nm as standard procedure to indicate that DNA is sufficiently free of protein. Titrations were done automatically by using the CerkoLab microinjector at 25 °C and in the range 200–450 nm (Figs. S7–S10*,* respectively). Electronic absorption spectra were recorded after each addition of different amounts of individual biomolecule solutions.

The concentration of freshly prepared *CT*-DNA was calculated based on a value of absorbance at 258 nm (Fig. S11; SI) and based on the calibration curve [ɛ_DNA_ 6,600 (base pairs) M^−1.^cm^−1^]^45,46^. The *3,6-PIRAMICAR* was dissolved in Tris–HCl to give 10 mL of the solution at mass concentration equal 2.36·10^–4^ M, which was used as an initial and pure carbazole derivative sample for both types of titration.

A 1.5 mL solution containing the appropriate concentration of *3,6-PIRAMICAR* was titrated by successive additions of a 1.92·10^–5^ M stock solution of *Bovine Serum Albumin* (BSA). The concentration of freshly prepared BSA (titrant solution) was established based on the calibration curve obtained at 278 nm (Fig. S12; SI).

### Biological evaluation

#### Strains and growth conditions of microorganisms

The antifungal activity was determined using the commercial strains*: Candida albicans* (ATCC 10231), *Candida glabrata* (DSM 11226) and *Candida krusei* (DSM 6128) and clinical fluconazole-resistant isolates from patients of the Children’s Memorial Health Institute in Warsaw (strain numbers are given in brackets): *Candida glabrata* (465), *Candida krusei* (2), *Candida krusei* (35), *Candida krusei* (74), *Candida krusei* (176) collected in years 2011 and 2012. Stock cultures of all strains were stored at − 80 °C with 15% v/v glycerol as cryopreservative. In each experiment, the yeast was subcultured on YPD agar plates (1% yeast extract, 2% peptone,2% glucose, and 2% agar) at 37 °C for 24 h, then inoculated in RPMI 1640 medium (Corning) supplemented with 1 M MOPS (3-N morpholinopropane sulfonic acid, EMD Chemicals) for 24 h at 37 °C under agitation at 180 rpm. After fungi incubation, inoculum amounting 10^5^ CFU/ml were added on YPD agar plates or RPMI 1640 medium, both buffered to pH 4.0, 5.5, or 7.0 with hydrochloric acid. Unless stated otherwise, all media and consumables were purchased from Sigma-Aldrich.

#### Antifungal activity

The antifungal activity was determined by the broth dilution method according to guidelines of the Clinical and Laboratory Standards Institute (M27-A3 document). RPMI 1640 medium was used for preparing serial dilutions of the test and reference compound using the 96-well microtiter plates. The final concentration of the solvent did not exceed 2.5% for DMSO and did not affect the growth of microorganisms. The inoculum amounting 10^5^ CFU/ml of all studied microorganism prepared from 24 h cultures of fungi grow at 37 °C were added to each dilution in a 1:1 ratio. Minimum Inhibitory Concentration (MIC) was established visually as the lowest concentration where no growth was observed after incubation for 24 h at 37 °C. To determine the lowest drug concentration that inhibits 50% of microorganisms’ growth, the microtitration plate was read with a microplate plate reader (Asys UVM 340 Microplate Reader, Biochrom). The absorbance was measured at 660 nm. Additionally, to obtain minimal fungicidal concentration (MFC), a 10 µl volume of suspension was taken from each well and transferred to a 48-well plate with YPD medium and incubated for 24 h at 37 °C. The MFC values were interpreted as the lowest concentration, where no growth of fungal was observed. Fluconazole was used as control antimicrobial agent.

#### Analysis of fungi by fluorescence microscopy

*Candida krusei* was incubated with the presence or absence of 10 µM *3,6-PIRAMICAR* on YPD agar plates or in broth RPMI 1640 medium, both buffered to pH 4 with hydrochloric acid for 24 h in 37 °C. Cell colonies from agar plates were transferred to the slide and imaged under × 20 magnification using a fluorescence microscope (IX83 Inverted Microscope, Olympus) connected to XC50 digital color camera (Olympus) with U-FGWA filter (Olympus). Cells in broth medium were washed three times in 0.05 mM potassium phosphate buffer (pH 7.4), centrifuge at 1,000 rpm for 5 min and fixed with 3.7% paraformaldehyde for 20 min at room temperature in the dark. Cells were washed two times with potassium phosphate buffer, immersed in 90% glycerol v/v, and imaged following the same procedures described for solid medium.

#### Morphological changes of fungi

*Candida krusei* cells (10^5^ CFU/ml) were treated in RPMI 1,640 buffered with hydrochloric acid to pH 4 with either 10 µM *3,6-PIRAMICAR* or 250 µM fluconazole under the agitation of 180 rpm for 4 h at 37 °C. After incubation, the cells were harvested by centrifugation (1,000 rpm, 5 min 4 °C) and resuspended in 0.05 mM potassium phosphate buffer (pH 7.0). Morphological changes of cells induced by treatment were assessment by illuminating cells by 480 nm light from an argon ionic laser and determining their position on a forward scatter (FSC) versus side scatter (SSC) contour plot using the Guava easyCyte 8 flow cytometer (Merck Millipore).

#### The change in fungal membrane permeability

Fungal membrane permeabilization was determined using the propidium iodide influx assay*. Candida krusei* cells (10^5^ CFU/ml) were treated with either 10 µM *3,6-PIRAMICAR* or 250 µM fluconazole in RPMI 1640 buffered with hydrochloric acid to pH 4 under the agitation of 180 rpm for 4 h at 37 °C. Cells were harvested by centrifugation (1,000 rpm, 5 min, 4 °C), washed in 0.05 mM potassium phosphate buffer (pH 7.4), and stain in the dark with 10 µM propidium iodide for 15 min at room temperature. A stock solution of 1.0 mg of PI per ml was prepared in deionized water and stored protected from light in the refrigerator. The uptake of propidium iodide into *C. krusei* cells was detected using a Guava easyCyte 8 flow cytometer (Merck Millipore) and analyzed by FlowJo v10 software.

## Conclusions

In summary, the complete structural, physicochemical and microbiological *3,6-PIRAMICAR* profile together with its interactions ability against potent cellular targets were reported in details. The received data enable conditions optimization (pH 4), which lead to achieving the confirmed *3,6-PIRAMICAR* activity against *Candida krusei*. The fungal cells membranes destruction observed in tests with *3,6-PIRAMICAR* is related to its lipophilic character (logP above 1.1)*.* In contrast to studies with fluconazole (representant of azoles drugs), the stimulation of *3,6-PIRAMICAR* antifungal activity was noted after the decrease of pH values in systems studied. Additionally, *3,6-PIRAMICAR* turn out to be more active against *Candida* species compared to its structural precursors, 3,6-diamine-9H-carbazole (3,6-NH_2_) and 2-amino-5-bromo-3-methylamine-pyrazine, respectively. Interestingly, the time-dependent experiments data were analyzed to note some subtle similarities between antifungal action established for *3,6-PIRAMICAR* and 3,6-NH_2_.

This paper demonstrates also the *3,6-PIRAMICAR* interaction possibilities with *CT*-DNA and BSA by using multi-experimental techniques. The compound studied shows a higher ability to bind BSA (1.9·10^5^ M^−1^) than with DNA (4.3·10^3^ M^−1^), respectively. The experimental results also indicate that a stable adduct is formed between the synthesized compound and proteins with dynamic quenching. As a perspective, we believe that our results can be considered as an initial for the research of executing the detailed mechanism of *3,6-PIRAMICAR* antifungal action.

### Supplementary information

Supplementary information.
