# Addendum: Photosensitive and pH-dependent activity of pyrazine-functionalized carbazole derivative as promising antifungal and imaging agent

**DOI:** 10.1038/s41598-024-59804-y

**Published:** 2024-05-15

**Authors:** Agnieszka Chylewska, Aleksandra M. Dąbrowska, Sandra Ramotowska, Natalia Maciejewska, Mateusz Olszewski, Maciej Bagiński, Mariusz Makowski

**Affiliations:** 1 Faculty of Chemistry, University of Gdańsk, Wita Stwosza 63, 80-308 Gdańsk, Poland; 2 Faculty of Chemistry, Gdańsk University of Technology, Gabriela Narutowicza 11/12, 80-233 Gdańsk, Poland

Addendum to: *Scientific Reports* 10.1038/s41598-020-68758-w, published online 16 July 2020

Following publication of this Article, concerns have been raised about the quality of the Supplementary Figures S21, S25, and S26. The mass spectra included in these figures are of resolution insufficient to clearly confirm the peak assignments. This is due to the software tool used for preparation of these figures.

The low-quality image of mass spectra in Figure S21 is due to poor print quality of the mass analysis result obtained in paper form, whereas for the proton (Figure S25) and carbon (Figure S26) NMR spectra, it is due to their being acquired with basic SpinWorks software, due to COVID-19 lock-down mobility issues. It also resulted in the incorrect representation of some peak assignments.

In this addendum, for Figure S21, authors provide an additional MALDI analysis (Figure 1) for 3,6-PIRAMICAR in the zoomed-in 410–460 m/z range (positive mode). This serves as evidence of the identity of the required and presented molecular mass values ([M + H +] found: 442.236). For Figures S25 and S26, authors employ licensed TopSpin 4.2.0 software to generate better quality Figures 2 and 3. This software employs different baseline correction methods and noise filtering techniques to provide better quality images.

**Figure 1:**
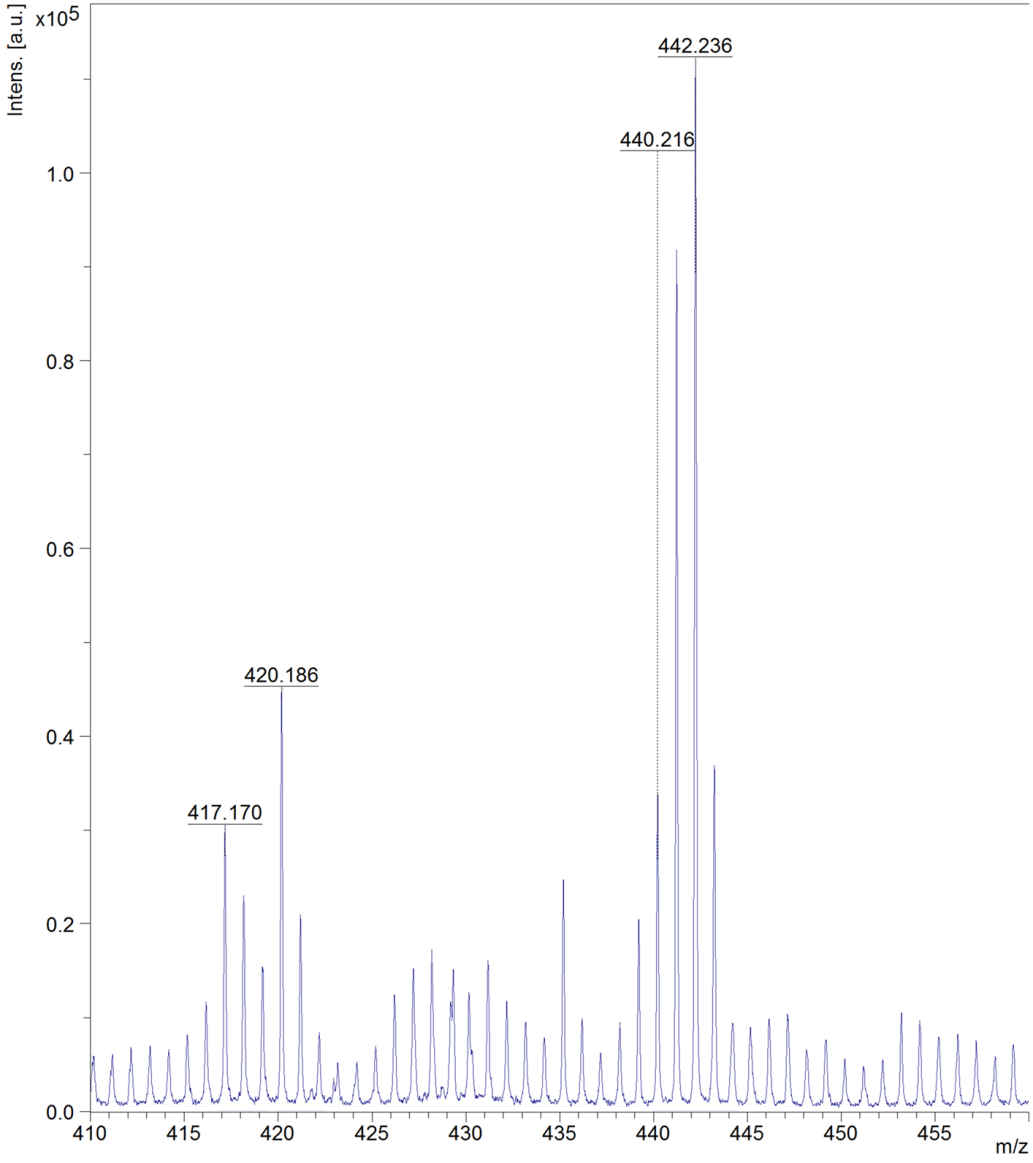
The corrected version of Fig. S21.

**Figure 2:**
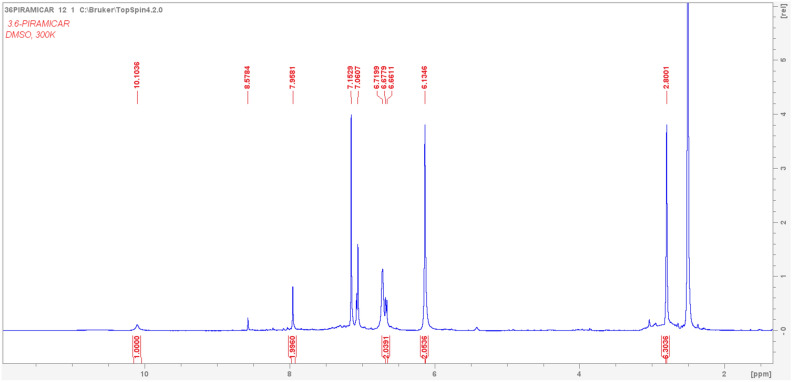
The corrected version of Fig. S25.

**Figure 3:**
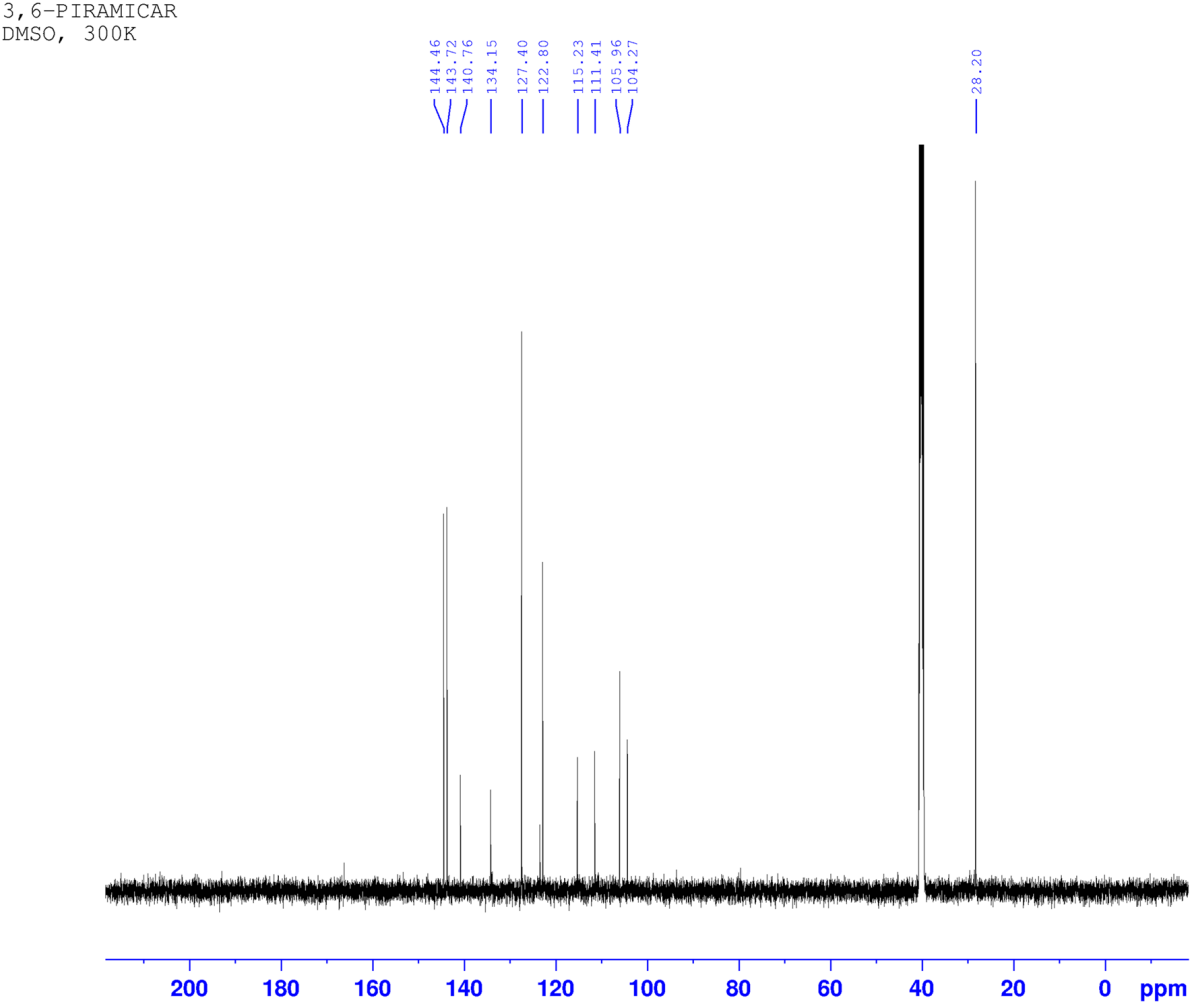
The corrected version of Fig. S26.

