# Supplementary material for: Photosensitive and pH-dependent activity of pyrazine-functionalized carbazole derivative as promising antifungal and imaging agent
Source: Sci Rep. 2020 Jul 16;10:11767. doi: 10.1038/s41598-020-68758-w (PMC7367338; doi:10.1038/s41598-020-68758-w)
Supplement: Supplementary file 1 — Supplementary information. [file 41598_2020_68758_MOESM1_ESM.docx]

***Supplementary Information***

***Photosensitive and pH-dependent Activity of Pyrazine-Functionalized Carbazole Derivative as Promising Antifungal and Imaging Agent***

Agnieszka Chylewska^1,^*, Aleksandra M. Dąbrowska^1^, Sandra Ramotowska^1^,

Natalia Maciejewska^2^, Mateusz Olszewski^2^, Maciej Bagiński^2^, Mariusz Makowski^1,^*

*^1^Faculty of Chemistry, University of Gdańsk, Wita Stwosza 63, 80-308 Gdańsk, Poland,*

*^2^Faculty of Chemistry, Gdańsk University of Technology, Gabriela Narutowicza 11/12, 80-233 Gdańsk, Poland*

*Corresponding authors: [mariusz.makowski@ug.edu.pl](about:blank); agnieszka.chylewska@ug.edu.pl

***Potentio- and UV–Vis spectrophotometric titration methods***

The spectro-pH-metric measurements for determination of the exact concentrations of HClO_4_ and NaOH stock solutions used for the spectrophotometric titrations were carried out at 25.0 ± 0.1 °C in aqueous solutions at a constant ionic strength in order to keep the activity coefficients constant. All the titrations were performed with carbonate-free NaOH solutions of known concentration. A CerkoLab pH-meter equipped with an InLab combined electrode and a syringe was used for the pH-metric titrations. The electrode system was calibrated using a HANNA instruments pH buffers set (pH: 1.00, 4.01, 6.00, 7.01, 9.00, 10.01) and a calibration curve was fitted to determine the Nernst slope (S) and E° correction factors. The above-mentioned analytical procedures were also described in our previous report [R1] and were treated by us as a standard methodology of these types measurement. Samples were deoxygenated by bubbling purified argon for ca. 10 min prior to the measurements, and argon was also passed over the solutions during further titrations. The Evolution 300 spectrophotometer UV–Vis double beam spectrophotometer, with an automatic stirrer, was used for absorbance measurements in the 200–700 nm interval. The path length was 1 cm. The spectrophotometric titrations were performed on samples of *3,6-PIRAMICAR* with the initial volume of the samples 1.0 mL. Measurements for the studied compound in HClO_4_ to NaOH system were also carried out by preparing individual samples in which NaOH partially or entirely replaced all acids. The above-mentioned analysis of pKa is based on the Henderson-Hasselbalch equation (1) [R1] in exponential form and was used to determine three pKa values for compound studied in aqueous solution (1.02 < pH < 11.90).

$\text{A = }\frac{\text{A}_{\text{1}}\text{+}\text{A}_{\text{2}}\text{∙}\text{10}^{\text{(pH-p}\text{Ka}_{\text{1}}\text{)}}}{\text{10}^{\text{(pH-p}\text{Ka}_{\text{1}}\text{)}}\text{+1}}\text{ + }\frac{\text{A}_{\text{2}}\text{+}\text{A}_{\text{3}}\text{∙}\text{10}^{\text{(pH-p}\text{Ka}_{\text{2}}\text{)}}}{\text{10}^{\text{(pH-p}\text{Ka}_{\text{2}}\text{)}}\text{+1}}\text{ + }\frac{\text{A}_{\text{3}}\text{+}\text{A}_{\text{4}}\text{∙}\text{10}^{\text{(pH-p}\text{Ka}_{\text{3}}\text{)}}}{\text{10}^{\text{(pH-p}\text{Ka}_{\text{3}}\text{)}}\text{+1}}$ (1)

The spectrophotometric titration by the Hammett acidity function concept was performed for an appropriate amount of *3,6-PIRAMICAR,* which was dissolved in diluted perchloric acid to obtain 10^-5^ M stock solution and by adding small amounts of concentrated (11.62 M; 70%) perchloric acid (25.0 ± 0.1°C). UV spectra were recorded for this method in the range of 200-500 nm with 1 cm matched quartz cells. The perchloric acid probe with the same concentration as that containing the studied compound solution was used as a reference solution. HClO_4_ was tested for optical clarity over the wavelength range used. Using the absorption spectra obtained for this method, estimations of three acidity constants of the studied compound were affected using modified expression (2), with pH replaced by *H_0_* parameter.

$\text{A = }\frac{\text{A}_{\text{1}}\text{+}\text{A}_{\text{2}}\text{∙}\text{10}^{\text{(}\boldsymbol{H}_{\boldsymbol{0}}\text{-p}\text{Ka}_{\text{1}}\text{)}}}{\text{10}^{\text{(}\boldsymbol{H}_{\boldsymbol{0}}\text{-p}\text{Ka}_{\text{1}}\text{)}}\text{+1}}\text{ + }\frac{\text{A}_{\text{2}}\text{+}\text{A}_{\text{3}}\text{∙}\text{10}^{\text{(}\boldsymbol{H}_{\boldsymbol{0}}\text{-p}\text{Ka}_{\text{2}}\text{)}}}{\text{10}^{\text{(}\boldsymbol{H}_{\boldsymbol{0}}\text{-p}\text{Ka}_{\text{2}}\text{)}}\text{+1}}\text{+ }\frac{\text{A}_{\text{3}}\text{+}\text{A}_{\text{4}}\text{∙}\text{10}^{\text{(}\boldsymbol{H}_{\boldsymbol{0}}\text{-p}\text{Ka}_{\text{3}}\text{)}}}{\text{10}^{\text{(}\boldsymbol{H}_{\boldsymbol{0}}\text{-p}\text{Ka}_{\text{3}}\text{)}}\text{+1}}$ (2)

Protonation constants and the individual spectra of the species were calculated by the computer program EQUID [R2-R4]. The calculations were always made from the experimental titration data measured in the absence of any precipitate in the solution. The obtained data were then analyzed using multi-wavelength analysis and nonlinear least squares regression method inputted in OriginLab software.


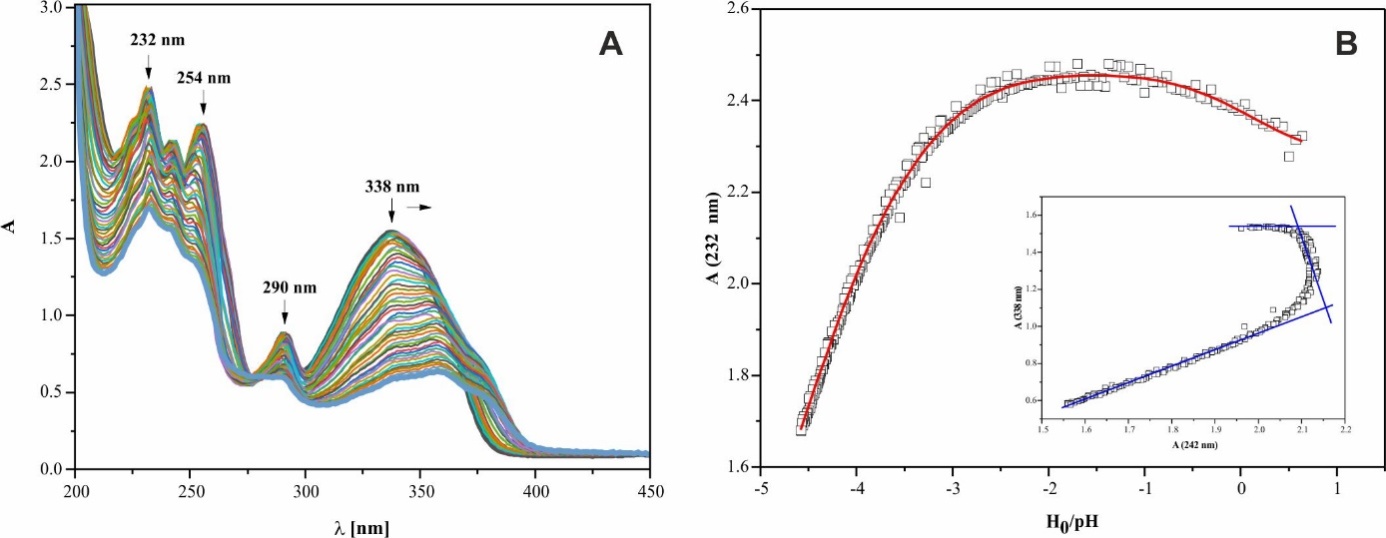


*Figure S1*. **A.** The titration spectral curves obtained for *3,6-PIRAMICAR* by using concentrated (70%) perchloric acid (red line is started and blue line is finished solutions); **B.** Plot of *H*_0_ against absorbance at 232 nm presented as data measured (black scatter) and fitting (red line); R^2^ = 0.996. Inset: plot of absorbances to show the relationship between absorbances at different wavelengths (A-diagram); the arrows (presented on **A**) show the direction of change upon the increase of perchloric acid concentration.

**
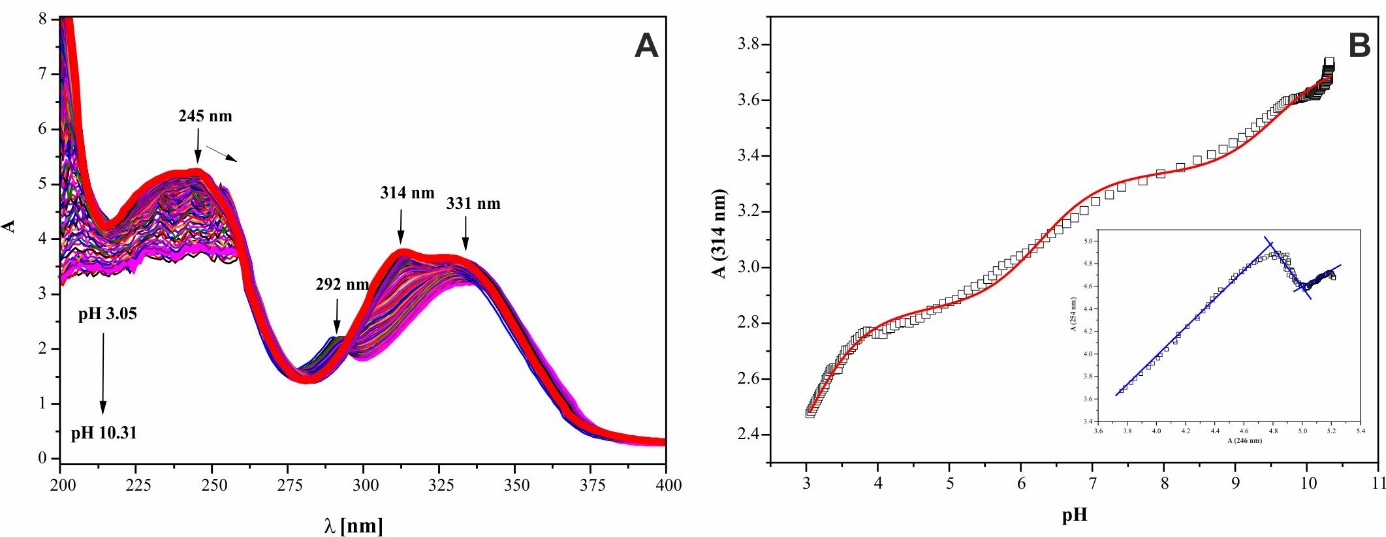
**

*Figure S2*. **A.** Change of the absorption spectra of *3,6-PIRAMICAR* with increasing pH from 3.00 to 10.31; started (red line) and finished (purpure line) solutions; **B.** Plot of pH against absorbance at 314 nm presented as data measured (black scatter) and fitting (red line); R^2^ = 0.991. Inset: plot of absorbances to show the relationship between absorbances at different wavelengths (A-diagram); the arrows (presented on **A**) show the direction of change upon the increase of pH.


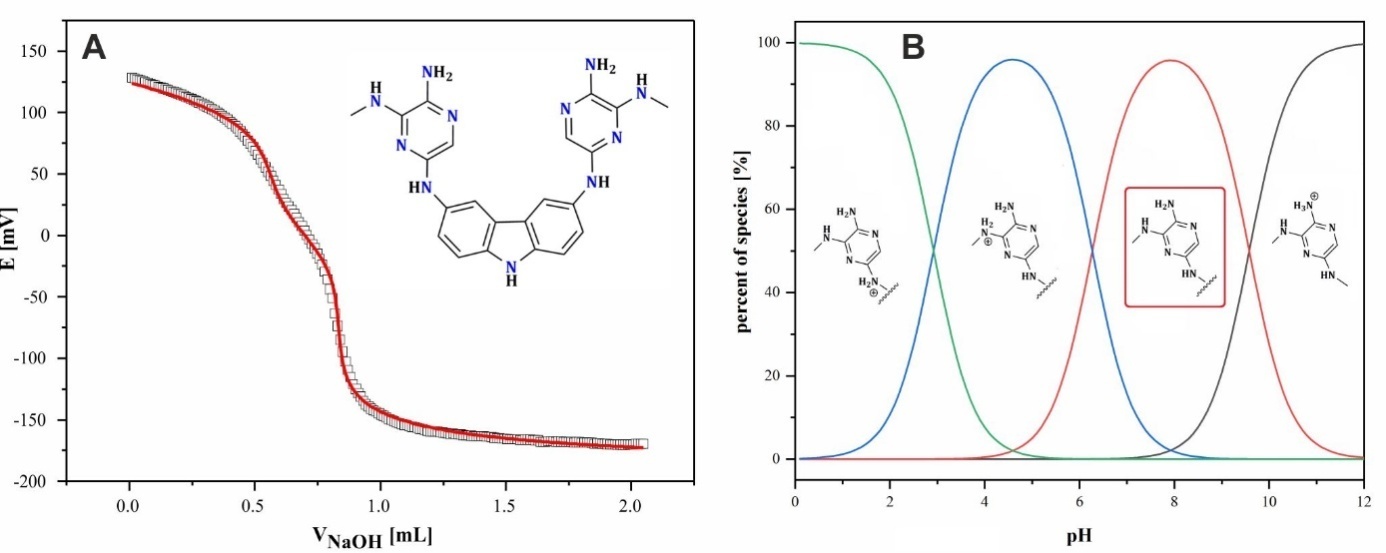


*Figure S3*. Potentiometric titration results: **A**. measured (black scatter) and calculated (red line) data of acidified *3,6-PIRAMICAR* solution; (R^2^ = 0.995). **B**. Distribution diagrams of *3,6-PIRAMICAR* species established in aqueous solution at different pH. In frame is presented neutral form of *3,6-PIRAMICAR.*

***Redox profile***





*Figure S4.* Voltamograms of **a)** 3,6-diaminocarbazole; **b)** 2-amino-5-bromo-3-(methylamino)pyrazine in acetonitrile.


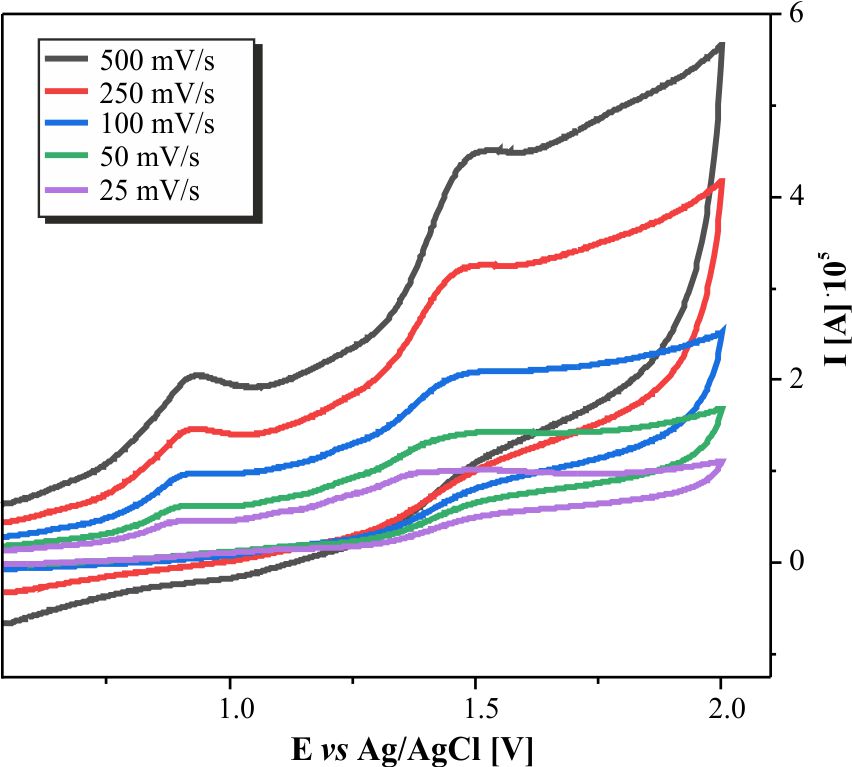


*Figure S5.* Voltammograms of *3,6-PIRAMICAR* at different scanning rates in acetonitrile.


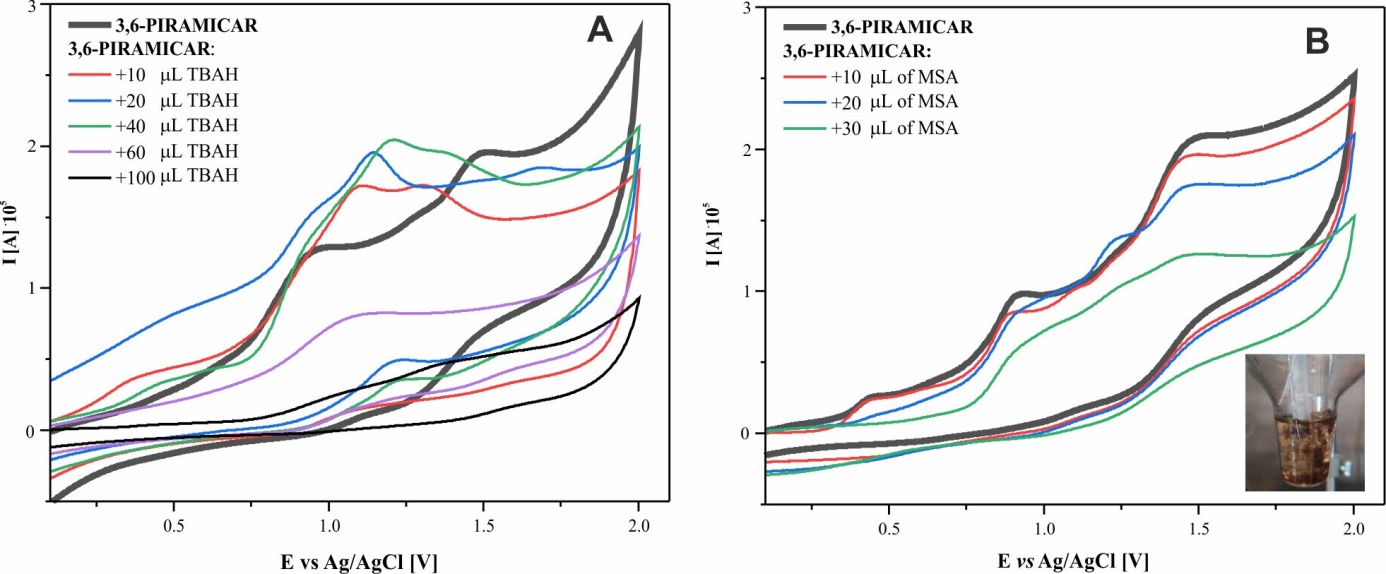


*Figure S6*. *3,6-PIRAMICAR* electrode processes registered as a result of additional amounts of: **A.** tetrabutylammonium hydroxide (TBAH); **B.**  methanesulfonic acid (MSA).


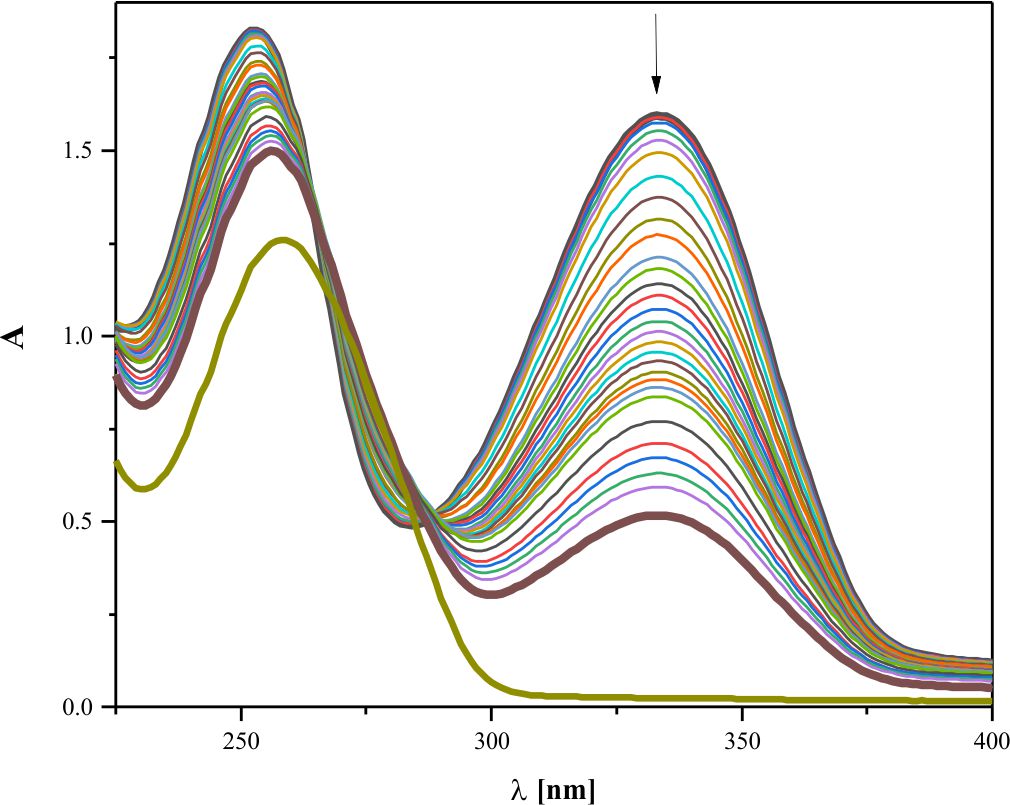


*Figure S7.* The complete spectra of *3,6-PIRAMICAR*-DNA titration system studied.


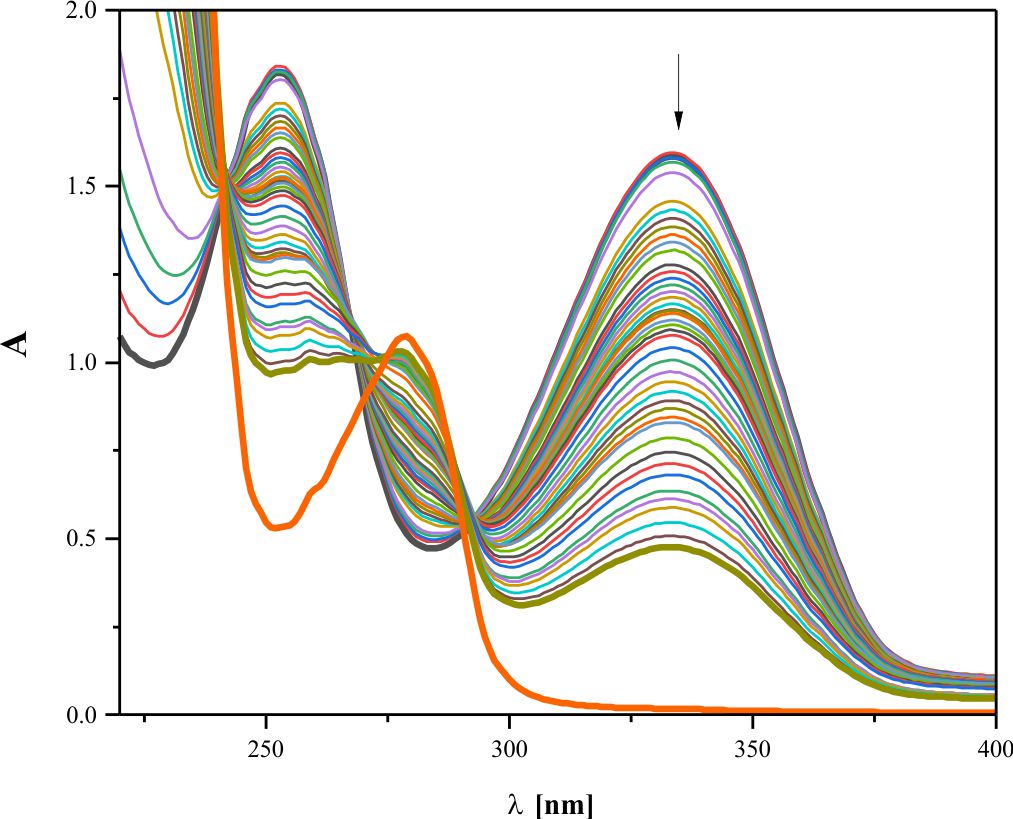


*Figure S8.* The complete spectra of *3,6-PIRAMICAR*-BSA titration system studied.

*
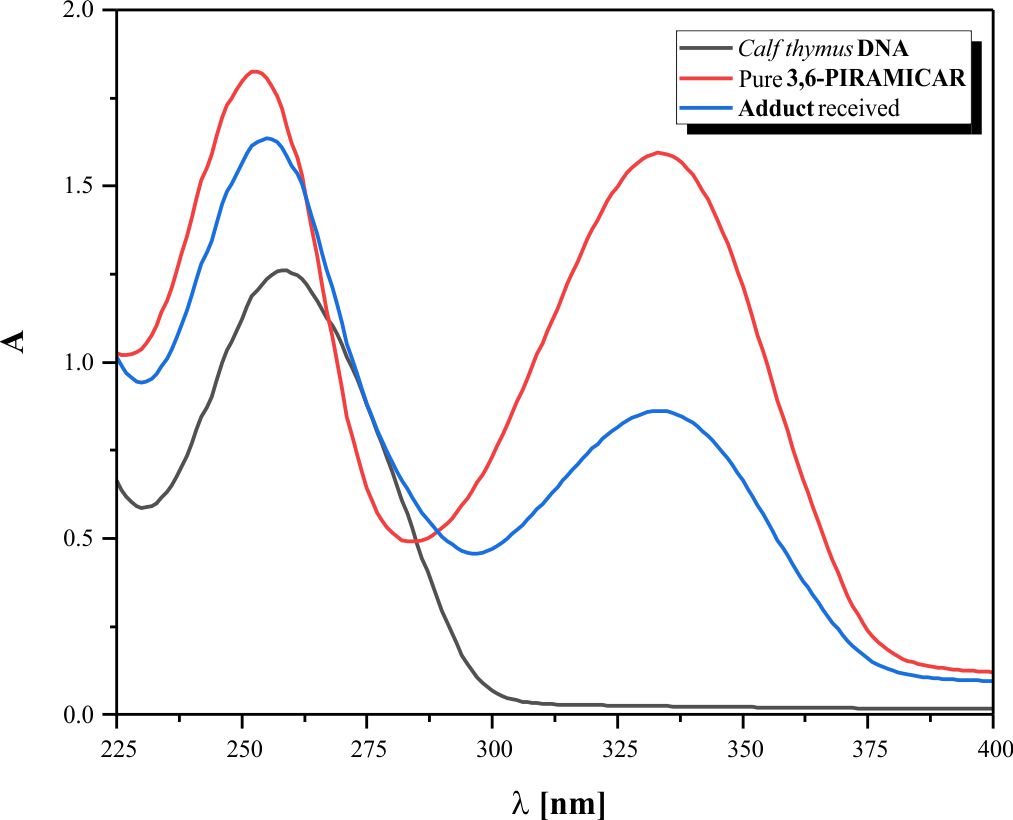
*

*Figure S9.* The comparison of individuals spectra of *3,6-PIRAMICAR*-DNA system studied.

*
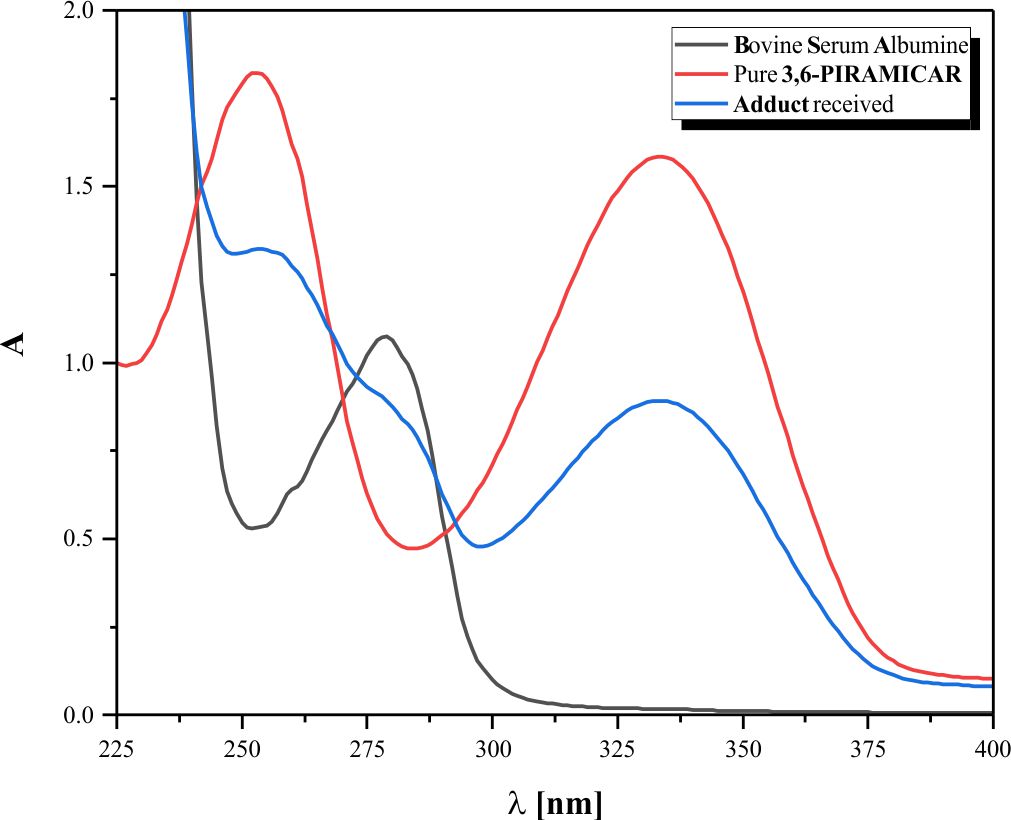
*

*Figure S10.* The comparison of individuals spectra of *3,6-PIRAMICAR*-BSA system studied.


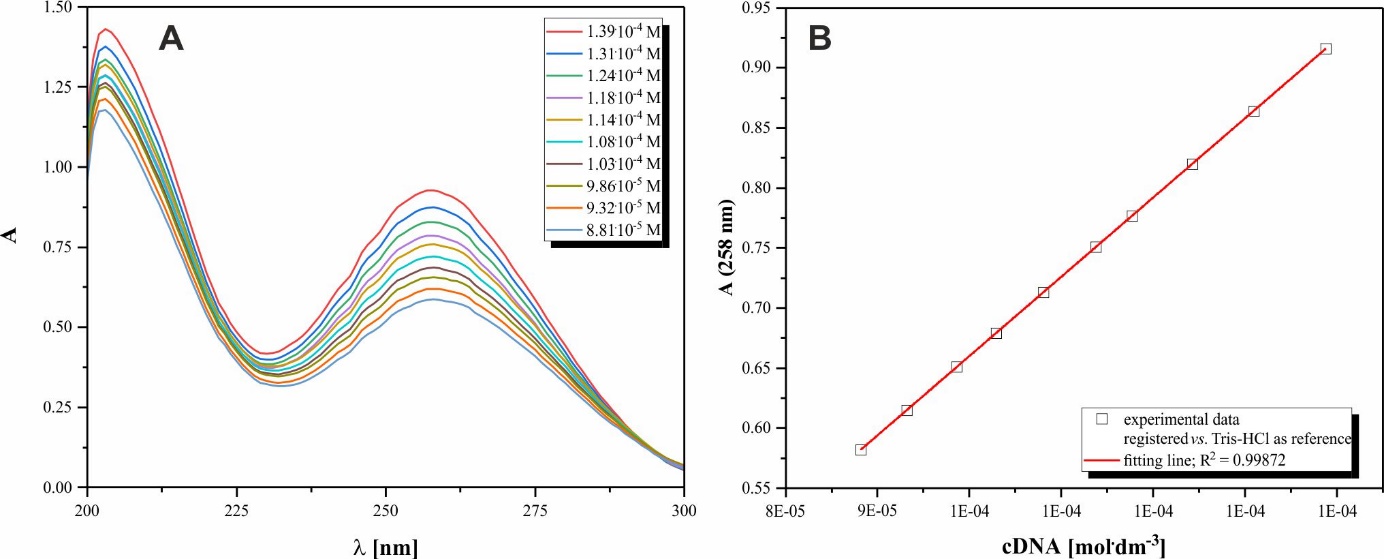


*Figure S11.* The determination of concentration for freshly prepared *CT*-DNA solutions **(A)** and the calibration curve **(B)**.

*
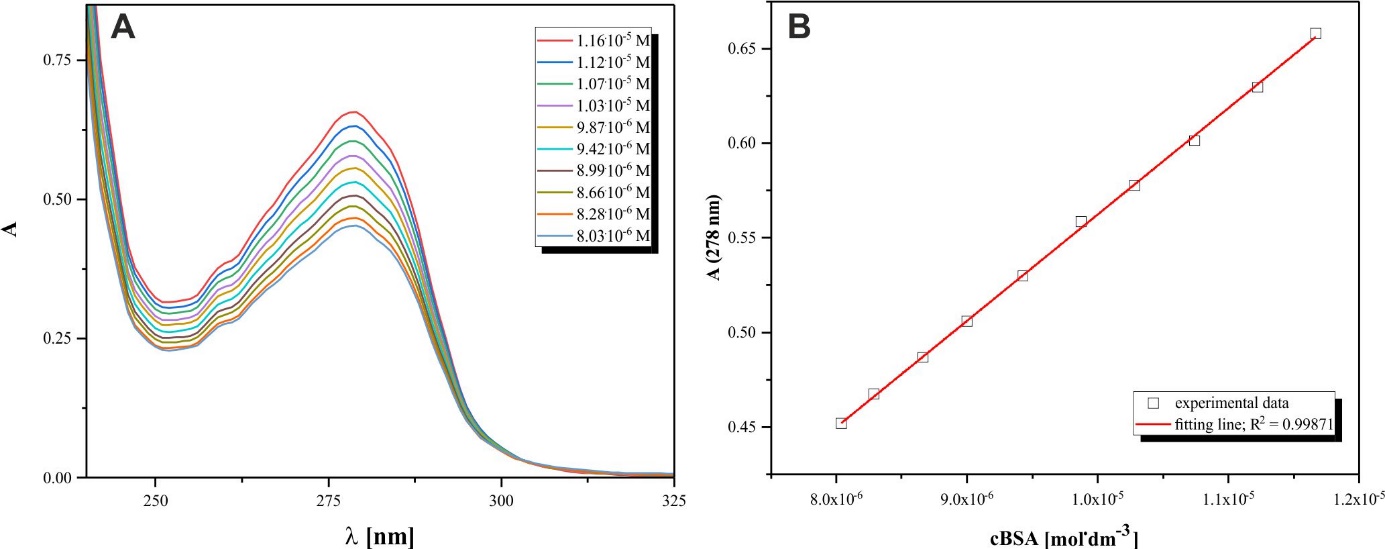
*

*Figure S12.* The determination of concentration for freshly prepared BSA solution.

**Biological evaluation**

*Anticancer activity*

Non-small cell lung cancer line A549 was obtained from ATCC (CCL-185) and was cultured in RPMI-1640 medium. Human colorectal cancer line HCT116 was kindly provided by Prof. Bert Vogelstein (Johns. Hopkins University, Howard Hughes Medical Institute, USA) and was grown in McCoy's5A medium. For both cancer cell lines, the culture medium was supplemented by 10% fetal bovine serum, 2 mM L-glutamine, and antibiotics (penicillin 62.6 µg/ml and streptomycin 40 µg/ml) at humidified atmosphere containing 5% CO_2_ at 37°C and routinely screened for Mycoplasma contamination. All reagents used to measure anticancer activity, unless stated otherwise, were purchased from Corning. Evaluation of cell viability was performed by 3-(4,5-dimethylthiazol-2-yl)-2,5-diphenyltetrazolium bromide (MTT, Sigma Aldrich) assay based on the metabolic ability of active cells to convert the yellow MTT reagent into a blue formazan. The cancer cells (8.75·10^3^ cells/ml) were seeded onto each well of a 96-well plate with the respective media and incubated to adhere overnight in the conditions as described above. Cells were further incubated in the presence with various concentrations of the investigated compounds, ranging from 0.4-250 μM or DMSO (Merck) as a control (1% v/v) for 72 h. After that, MTT reagent (20 μl, 4 mg/ml) was added to each well and incubated for 2h at 37 °C. The medium was then discarded, and formazan dye formed was dissolved in DMSO. The absorbance was determined at 570 nm with a microplate reader (Asys UVM 340 Microplate Reader, Biochrom). All experiments were performed three times independently, each in triplicate.

*Antibacterial activity*

The antibacterial activity was determined using the following strains: *Staphylococcus aureus* (ATCC 25923), *Escherichia coli* (ATCC 25922) and *Bacillus cereus* (PCM 2003) by the ~~tube~~ serial dilution method according to guidelines of the Clinical and Laboratory Standards Institute (M07-A10 document). Mueller-Hinton broth medium was for preparing serial dilutions of the test and reference compounds using the 96-well microtiter plates. The inoculum amounting 10^5^ CFU/ml of all studied microorganisms prepared from 24 h cultures of bacteria grow at 37°C were added to each dilution in a 1:1 ratio. Minimum Inhibitory Concentration (MIC) was established visually as the lowest concentration where no growth was observed after incubation for 24 h at 37 °C. Ciprofloxacin and levofloxacin were used as control antimicrobial agents.

*Table S1.* Anticancer activity of investigated compounds defined as IC50 [µM].

| Compound | Tumor cell line | |
| --- | --- | --- |
|  | A549 | HCT116 |
| Mitoxantrone | 1.56 ± 0.08 | 0.025 ± 0.001 |
| ABMAP | >250 | >250 |
| 3,6-NH_2_ | >250 | >250 |
| 3,6-PIRAMICAR | >250 | >250 |

*Table S2.* Antifungal activities of compounds against clinical isolates of yeast (MIC, MIC50, MFC μM).

| Strain | pH | 3,6NH_2_ | | | *3,6-PIRAMICAR* | | | | Fluconazole | | |
| --- | --- | --- | --- | --- | --- | --- | --- | --- | --- | --- | --- |
|  |  | MIC50 | MIC | MFC | MIC50 | MIC | MFC | MIC50 | | MIC | MFC |
| *C. albicans* 4 | 7.0 | >250 | >250 | >250 | >250 | >250 | >250 | >250 | | >250 | >250 |
|  | 5.5 | >250 | >250 | >250 | >250 | >250 | >250 | >250 | | >250 | >250 |
|  | 4.0 | >250 | >250 | >250 | 195.34 ± 8.10 | >250 | >250 | >250 | | >250 | >250 |
| *C. glabrata* 465 | 7.0 | >250 | >250 | >250 | >250 | >250 | >250 | >250 | | >250 | >250 |
|  | 5.5 | >250 | >250 | >250 | >250 | >250 | >250 | >250 | | >250 | >250 |
|  | 4.0 | >250 | >250 | >250 | 140.85 ± 9.69 | 250 | >250 | >250 | | >250 | >250 |
| *C. krusei* 2 | 7.0 | >250 | >250 | >250 | >250 | >250 | >250 | >250 | | >250 | >250 |
|  | 5.5 | >250 | >250 | >250 | >250 | >250 | >250 | >250 | | >250 | >250 |
|  | 4.0 | >250 | >250 | >250 | 138.08 ± 23.33 | 250 | >250 | >250 | | >250 | >250 |
| *C. krusei* 35 | 7.0 | >250 | >250 | >250 | >250 | >250 | >250 | >250 | | >250 | >250 |
|  | 5.5 | >250 | >250 | >250 | >250 | >250 | >250 | >250 | | >250 | >250 |
|  | 4.0 | >250 | >250 | >250 | 135.06 ± 8.83 | 250 | 250 | >250 | | >250 | >250 |
| *C. krusei* 74 | 7.0 | >250 | >250 | >250 | >250 | >250 | >250 | 89.65 ± 12.74 | | >250 | >250 |
|  | 5.5 | >250 | >250 | >250 | >250 | >250 | >250 | 102.41 ± 26.56 | | 250 | >250 |
|  | 4.0 | >250 | >250 | >250 | 98.97 ± 20.53 | 250 | >250 | >250 | | >250 | >250 |
| *C. krusei* 176 | 7.0 | >250 | >250 | >250 | >250 | >250 | >250 | >250 | | >250 | >250 |
|  | 5.5 | >250 | >250 | >250 | >250 | >250 | >250 | >250 | | >250 | >250 |
|  | 4.0 | >250 | >250 | >250 | 111.21 ± 8.80 | 250 | >250 | >250 | | >250 | >250 |


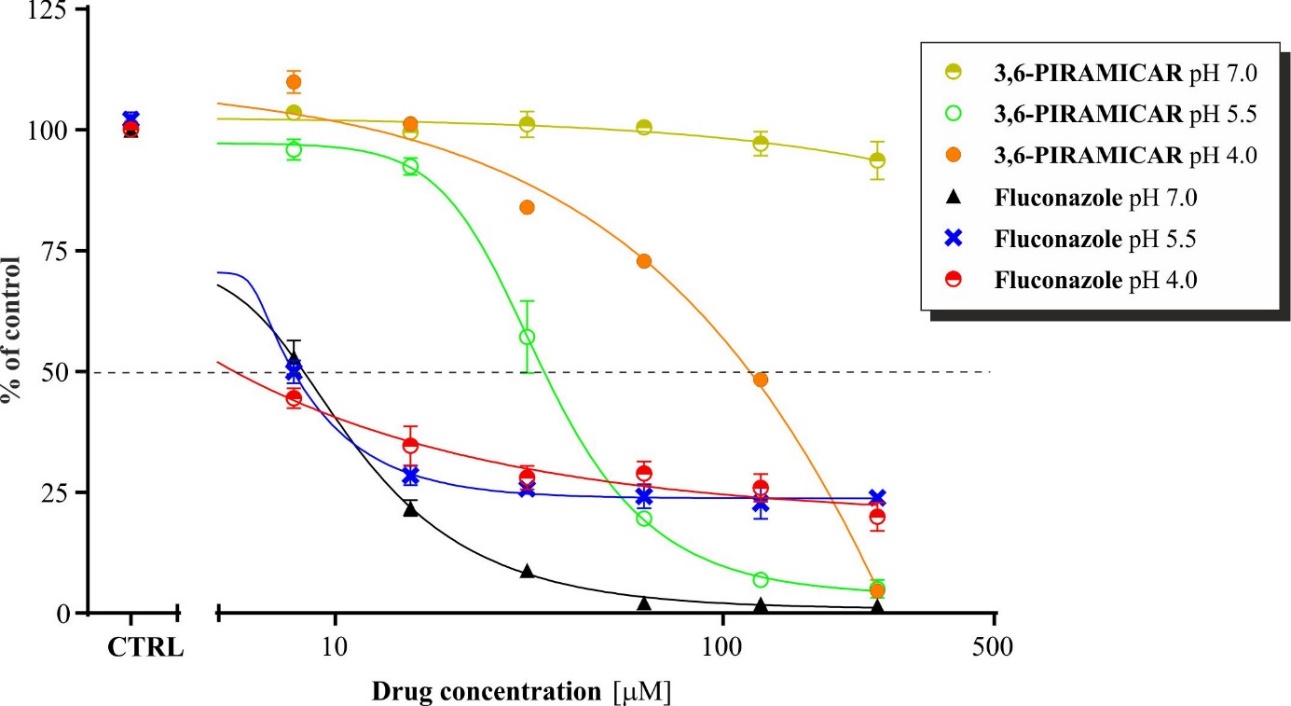


*Figure S13.* Comparison of *3,6-PIRAMICAR* and Fluconazole antifungal activity against *Candida albicans* at pH 7.0, 5.5 and 4.0.


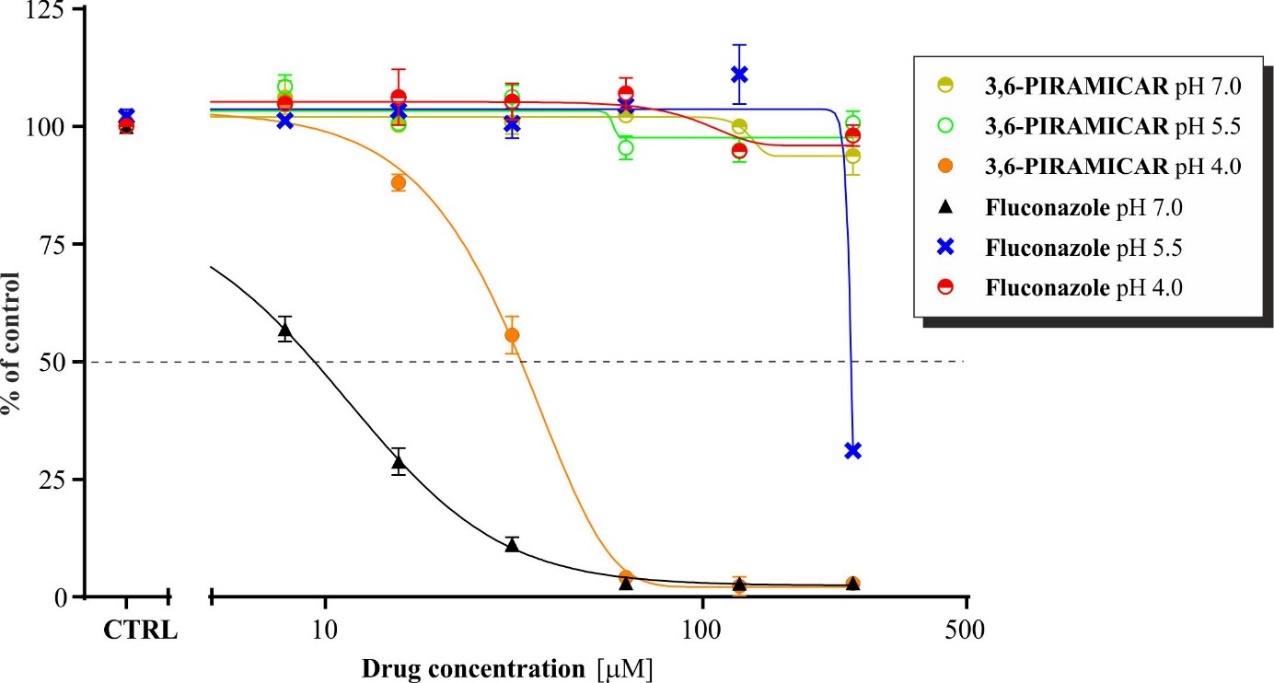


*Figure 14.* Comparison of *3,6-PIRAMICAR* and Fluconazole antifungal activity against *Candida glabrata* at pH 7.0, 5.5 and 4.0.

*
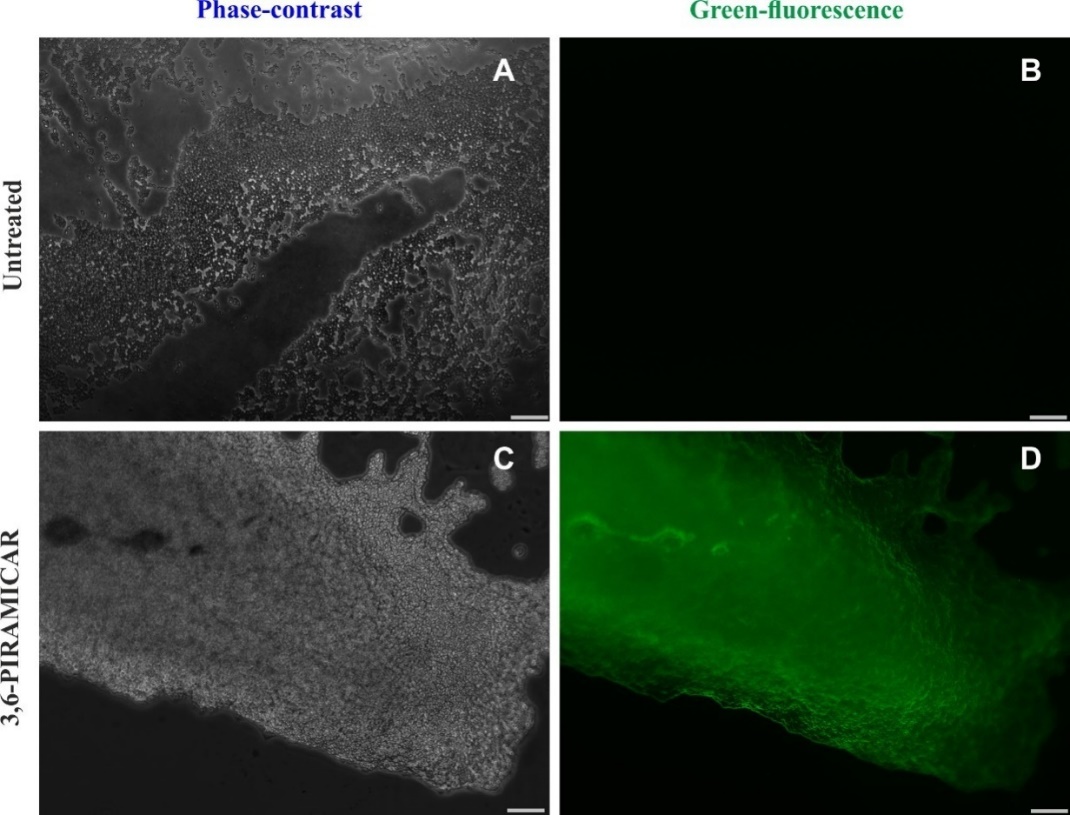
*

*Figure S15*. Fluorescent microscopy of *Candida krusei* cultures on agar plates after exposure to
*3,6-PIRAMICAR*. Scale bar = 50 μm.

*Table S3.* Antibacterial activity of investigated compounds defined as MIC [µM].

| Compound | Bacteria strain | | |
| --- | --- | --- | --- |
|  | Gram (+) | | Gram (-) |
|  | *Bacillus cereus* | *Staphylococcus aureus* | *Escherichia coli* |
| Ciprofloxacin | 0.25 | 0.02 | 0.005 |
| Levofloxacin | 0.25 | 0.02 | 0.01 |
| ABMAP | > 250 | > 250 | > 250 |
| 3,6-NH_2_ | > 250 | > 250 | > 250 |
| 3,6-PIRAMICAR | > 250 | > 250 | > 250 |

***Partition (P) and distribution (D) coefficients for 3,6-PIRAMICAR***

Firstly, calibration curves were prepared for substance studied. The curves relate the measured absorbance by the UV spectrophotometer to the actual concentration of the compound in aqueous solution. *3,6-PIRAMICAR* aqueous solution absorbs UV radiation at 250 and 334 nm. Both calibration curves for aqueous and octanol *3,6-PIRAMICAR* samples, as well as the confirmation of correlated with the law of absorption, were presented as *Figures S16* and *S17*. The linear relationship between absorbance and concentration is very high.


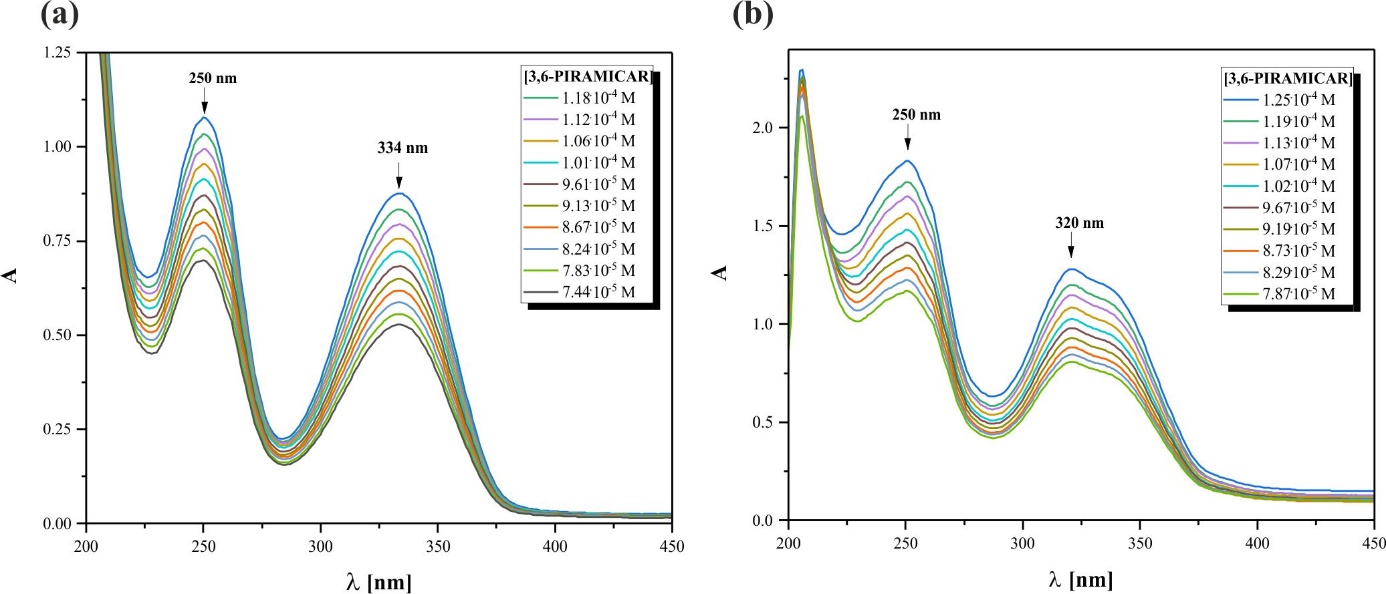


*Figure S16.* Lambert-Beer law as spectra of *3,6-PIRAMICAR* in: (a)aqueous and (b) octanol solutions.

*
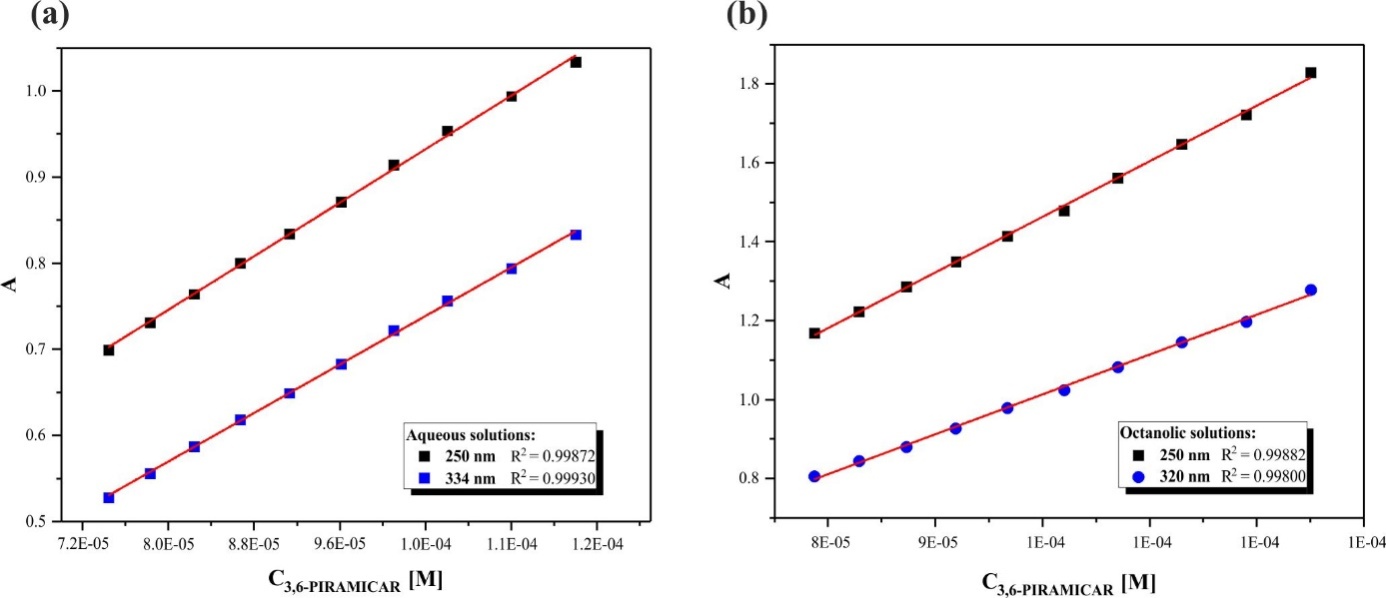
*

*Figure S17.* The relationships between absorbance and concentration of *3,6-PIRAMICAR* in: (a) aqueous and (b) octanol solutions.

The *3,6-PIRAMICAR* aqueous concentrations (C) is related to absorbance (A) by the following Eqs. (e1) at 250 nm and (e2) at 334 nm, but for *3,6-PIRAMICAR* octanol solutions – by Eqs. (e3) at 250 nm and (e4) at 320 nm:

A_W_ = 7774.95 · C_W_ + 0.12383 (e1)

A_W_ = 7045.73 · C_W_ + 0.00593 (e2)

A_OCT_ = 14096.13 · C_OCT_ + 0.05345 (e3)

A_OCT_ = 10105.38· C_OCT_ + 0.00253 (e4)

*
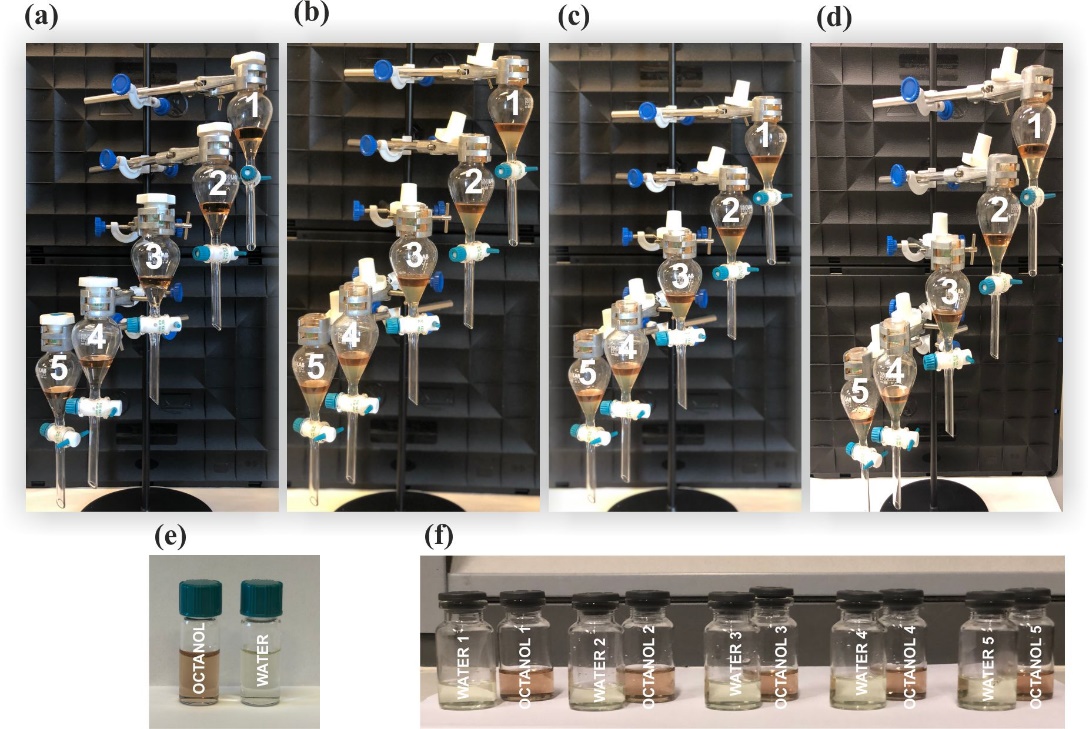
*

*Figure S18.* The course of research related to the extraction in the octanol/water (O/W) system: **(a)** the start moment (before extraction); **(b)** the samples directly after extraction (30 mins); **(c)** the division (after 2 hours of the extraction end); **(d)** the complete division (the second day after the extraction); **(e)** the colors of *3,6-PIRAMICAR* solutions (independent solutions of compound studied to comparison of colors); **(f)** separated phases received as a result of extraction.

These above expressions were used to calculate the unknown concentration of *3,6-PIRAMICAR* in aqueous and octanol fractions received as a result of extractions in the O/W system studied (*Figure S19*).

***
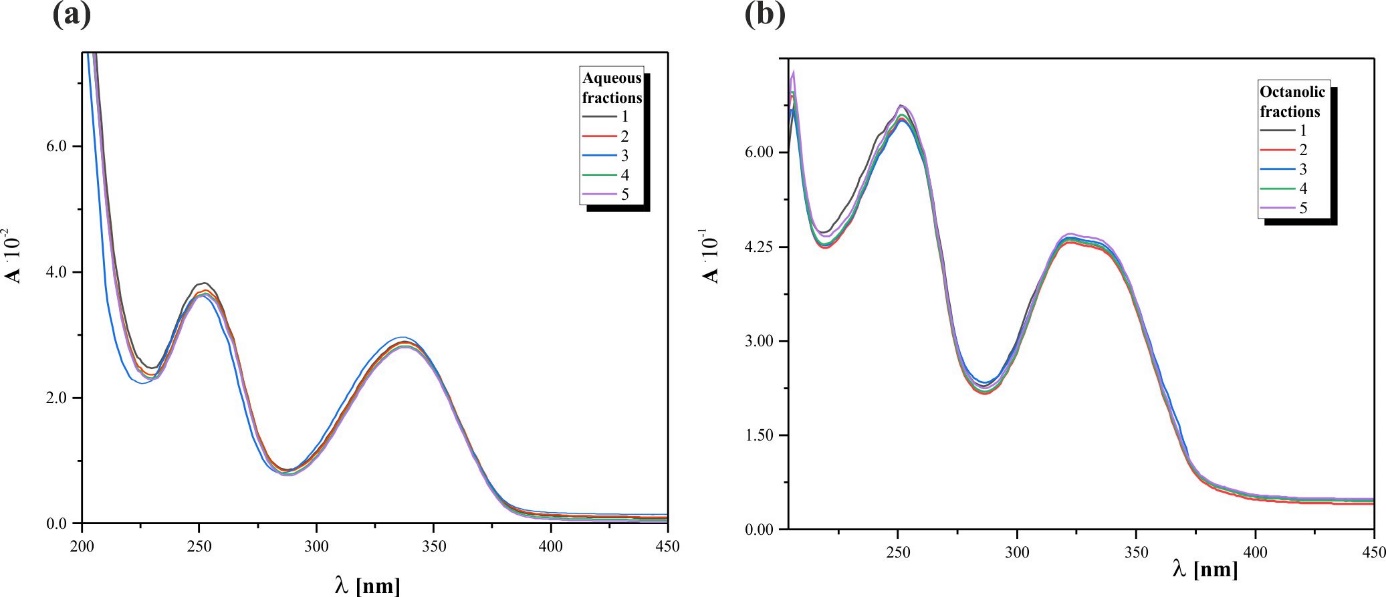
***

*Figure S19.* Electronic spectra obtained for aqueous **(a)** and octanol **(b)** fractions as a result of extractions (O/W system studied).

The individual values of equilibria concentrations received (C’) were collected together in *Table S4* for this experiment.

Moreover, the ‘n’ parameter, classified as the number of molecules associated or dissociated of solute molecules, in n-octanol as well as in water was checked and was equal to 3.85 (*Figure S20*). The experiment was performed at constant temperature (room temperature). *3,6-PIRAMICAR* concentrations values of five probes calculated for the octanol fractions were plotted against the corresponding *3,6-PIRAMICAR* equilibrium aqueous concentrations at 250 nm and 320 nm, respectively.

The ‘n’ parameter was established as a slope of the relationship between logC’_OCT_ and logC’_W_ and is 3.85 what confirmed that the *3,6-PIRAMICAR* studied forms 4 molecules association aggregate in the octanol medium.

*Table S4.* Experiment results (O/W): extractions data, partition and distribution coefficients for *3,6-PIRAMICAR* octanol solutions (c = 0.406mM; one probe to extraction was volume of 5 mL); the volume of pure water used to extraction of **P(1)**-**P(5)** was 5 mL; at room temperature.

| Fractions data obtained at 250 nm ^a)^ as a result of extractions | | | | |
| --- | --- | --- | --- | --- |
| **PROBE** | **A’_OCT_** | **A’_W_** | **C’_OCT_ [M ⋅10^-4^]** | **C’_W_ [M ⋅10^-5^]** |
| **P(1)** | 0.673 | 0.045 | 4.73 | 4.18 |
| **P(2)** | 0.658 | 0.045 | 4.63 | 4.16 |
| **P(3)** | 0.661 | 0.044 | 4.65 | 4.07 |
| **P(4)** | 0.664 | 0.045 | 4.67 | 4.08 |
| **P(5)** | 0.672 | 0.040 | 4.73 | 3.62 |
| **Probe** | **D** | **Log D** | **P** | **Log P** |
| **P(1)** | 0.946 | -0.024 | 11.31 | 1.05 |
| **P(2)** | 0.926 | -0.033 | 11.15 | 1.05 |
| **P(3)** | 0.930 | -0.032 | 11.41 | 1.06 |
| **P(4)** | 0.934 | -0.030 | 11.46 | 1.06 |
| **P(5)** | 0.946 | -0.024 | 13.09 | 1.12 |
|  | **D_av._** | **Log D_av._** | **P_av._** | **Log P_av._** |
|  | 0.94 | -0.028 | 11.68 | 1.07 |
| Fractions data obtained at 320 nm ^a)^ as a result of extractions | | | | |
| **PROBE** | **A’_OCT_** | **A’_W_** | **C’_OCT_**  **[M ⋅10^-4^]** | **C’_W_**  **[M ⋅10^-5^]** |
| **P(1)** | 0.462 | 0.024 | 4.57 | 4.49 |
| **P(2)** | 0.456 | 0.023 | 4.52 | 4.47 |
| **P(3)** | 0.467 | 0.023 | 4.62 | 4.47 |
| **P(4)** | 0.465 | 0.022 | 4.60 | 4.41 |
| **P(5)** | 0.473 | 0.022 | 4.68 | 4.41 |
| **Probe** | **D** | **Log D** | **P** | **Log P** |
| **P(1)** | 0.946 | -0.024 | 13.52 | 1.13 |
| **P(2)** | 0.927 | -0.032 | 13.88 | 1.14 |
| **P(3)** | 0.931 | -0.031 | 14.35 | 1.15 |
| **P(4)** | 0.935 | -0.029 | 14.85 | 1.17 |
| **P(5)** | 0.946 | -0.024 | 14.35 | 1.15 |
|  | **D_av._** | **Log D_av._** | **P_av._** | **Log P_av._** |
|  | 0.94 | -0.028 | 14.19 | 1.15 |

^a)^calibration curves and octanol/water phases spectra were included in *Figures S16* and  *S17*

**
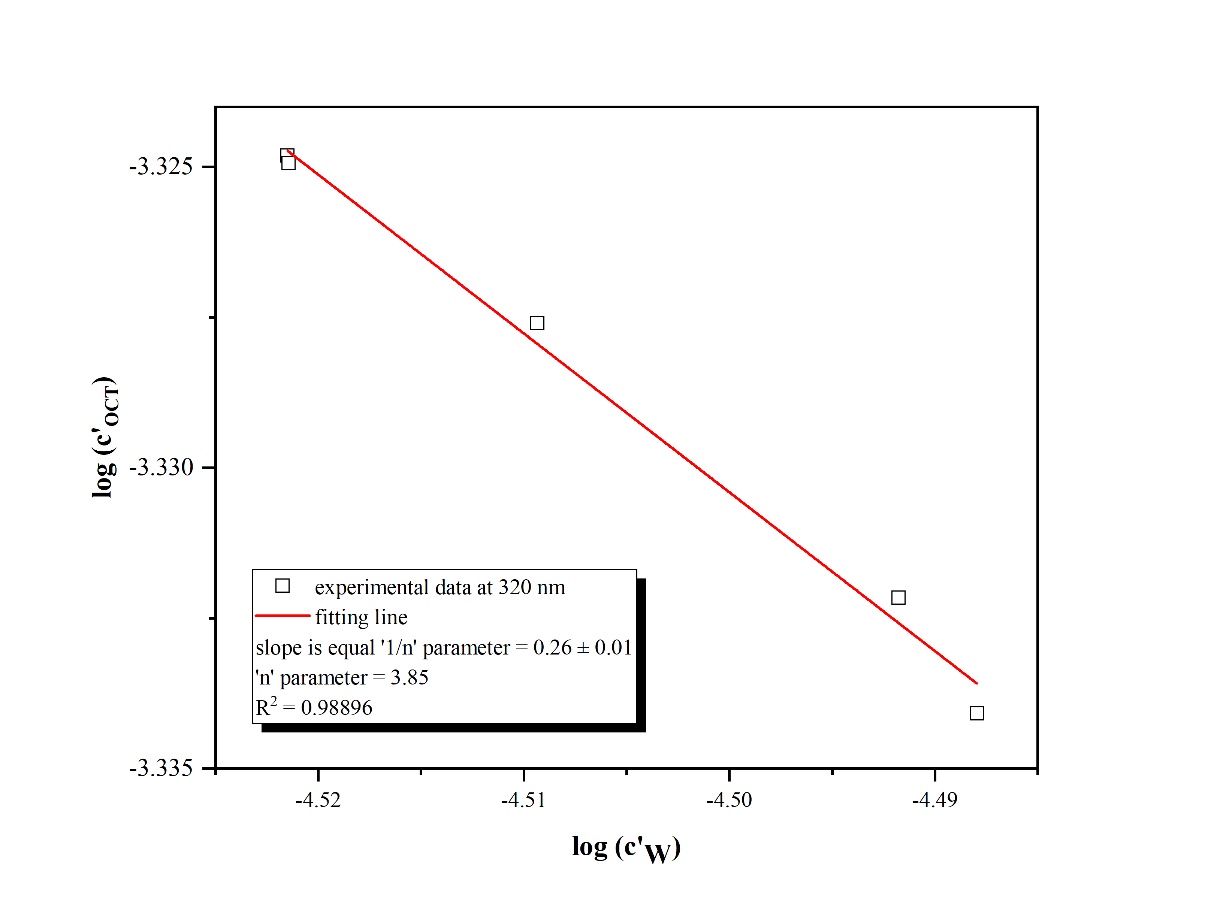
**

*Figure S20.* The graphical presentation of ‘n’ parameter which defines the number of *3,6-PIRAMICAR* molecules associated in octanol at 320 nm.

***Materials and reagents***

Unless otherwise indicated, all reagents were purchased from commercial resources (*Sigma-Aldrich* as well as *TCI America*) and used without further purification. Twice distilled water (Hydrolab-Reference purified) with conductivity not exceeding 0.09 µS/cm was used in the preparation of all the solutions under study.

***Synthetic procedures***

*3,6-diiodo-9-N-tosylcarbazole* **(1)**

3,6-diiodo-9H-carbazole (3.58 mmol, 1.5 g) and KOH (16.4 mmol, 0.92 g) in acetone (20 mL) was slowly added to a solution of tosylCl (16.4 mmol, 3.127 g) dissolved in 2 mL of acetone. The mixture was refluxed for 15 minutes, then poured into water (50 mL) and extracted by CH_2_Cl_2_. The organic phase received was washed by water and dried over anhydrous MgSO_4_. After concentration, the light-yellow **1** was obtained (1.35 g, 66%) as a result of recrystallization in the system of CH_2_Cl_2_/petroleum ether. ^1^H NMR (400 MHz, CDCl_3_) δ [ppm]: 8.35 (d, 2H), 8.07 (d, 2H),7.94 (dd, 2H), 7.44 (d, 2H), 7.28 (d, 2H), 2.51 (s, 3H).

*3,6-diamino-9-N-tosylcarbazole* **(2)**

To a solution of 1.18 g (4 mmol) Cu(NO_3_)_2_·6H_2_O in a mixture of CH_3_COOH/(CH_3_COO)_2_ (v/v=10/30 mL), 1.25 g (3 mmol) of **1** were introduced in small portions. The reaction mixture was stirred at room temperature for 1h and then poured on cooled water. In the next step, 2.25 g (10 mmol) SnCl_2_·2H_2_O,10 mL CH_3_COOH and 14 mL, 36% HCl were added to the mixture formed. The synthetic solution was refluxed under nitrogen atmosphere for 24 h. The initial yellow color turns brown during this time. After 24h, the mixture was neutralized with aqueous NaOH solution (15%), and the dark brown precipitate was separated by filtration, washed with water and dried over anhydrous MgSO_4_. The product was purified by using water and acetone. Yield = 1.21 g (38.1%). ^1^H NMR (400 MHz, CDCl_3_) δ [ppm]: 7.84 and 7.62 (d, C**H** arom. tosyl), 7.42 (d, C**H** arom. carbazole), 7.18 (d, C**H** arom. tosyl), 7.15 (C**H**), 6.10 (s, –N–C**H**_2_–), 2.51 (s, -C**H_3_** tosyl).

*3,6-di-(2-amino-3-methylaminopyrazino)-9-N-tosylcarbazole* **(3)**

2-amino-5-bromo-3-methylaminopyrazine ABMAP (6.3 mmol, 1.28 g), 3,6-diamino-9-N-tosylcarbazole (3 mmol, 1.05 g), CuI (6.3 mmol, 0.4 g), K_2_CO_3_ (6.5 mmol, 0.89 g) and toluene (45 mL) was stirred under nitrogen atmosphere at 180 ^o^C for 12h. The reaction mixture was poured into an excess of methanol to precipitate the product. The brown precipitate was collected by filtration and washed three times by methanol. The crude product was filtered and re-precipitated from the DMF/methanol solvent system to afford 1.49 g of brown powder of **3**.
^1^H NMR (400 MHz, CDCl_3_) δ [ppm]: 8.32, 8.138, 7.78 and 7.44 (d, C**H** arom. tosyl), 7.90 and 7.56 (d, C**H** arom. carbazole), 7.00 (C**H**), 6.64 (CH arom. pyrazine), 3.35 (s, -C**H_3_**), 2.51 (s, -C**H_3_** tosyl).

*3,6-di-(2-amino-3-methylaminopyrazino)-9-H-carbazole 3,6-PIRAMICAR* **(4)**

The **3** was dissolved in DMSO with addition of KOH amount (1.2 mmol, 68 mg) was kept at reflux for 90 minutes. The **4** was received as dark-violet powder (898 mg, 92%) as a result of addition a small portion of 10% HCl and recrystallization (CH_2_Cl_2_/methanol). Elemental analysis: calculated for C_22_H_23_N_11_, C; 59.85%, H; 5.25%, N; 34.90%; found, C; 60.09%, H; 5.59%, N; 34.52%. IR (ATR) ν [cm^-1^]: 3421, 3239,3155, 2938,1642, 1583, 1566, 1456, 1403, 1295, 1240, 1214, 1174, 1114, 1099, 1006, 921, 844, 825, 811, 779, 733, 700, 633, 588. ^1^H NMR (400 MHz, d^6^-DMSO) δ [ppm]: 10.10 (s, 1H), 8.57 (s, 2H), 7.97 (s, 2H), 7.15 and 7.04 (dd, 4H), 6.73 - 6.64 (s, 4H; d, 2H), 6.11 (s, 2H), 2.89 (s, 6H); ^13^C NMR (400 MHz, d^6^-DMSO) δ [ppm]: 144.47; 143.68; 140.68; 134.20; 127.41; 122.82; 115.24; 111.29; 107.3; 104.18; 28.02; (MS/TOF) *m/z* calculated [M+H]^+^ 442.21, found 442.20.


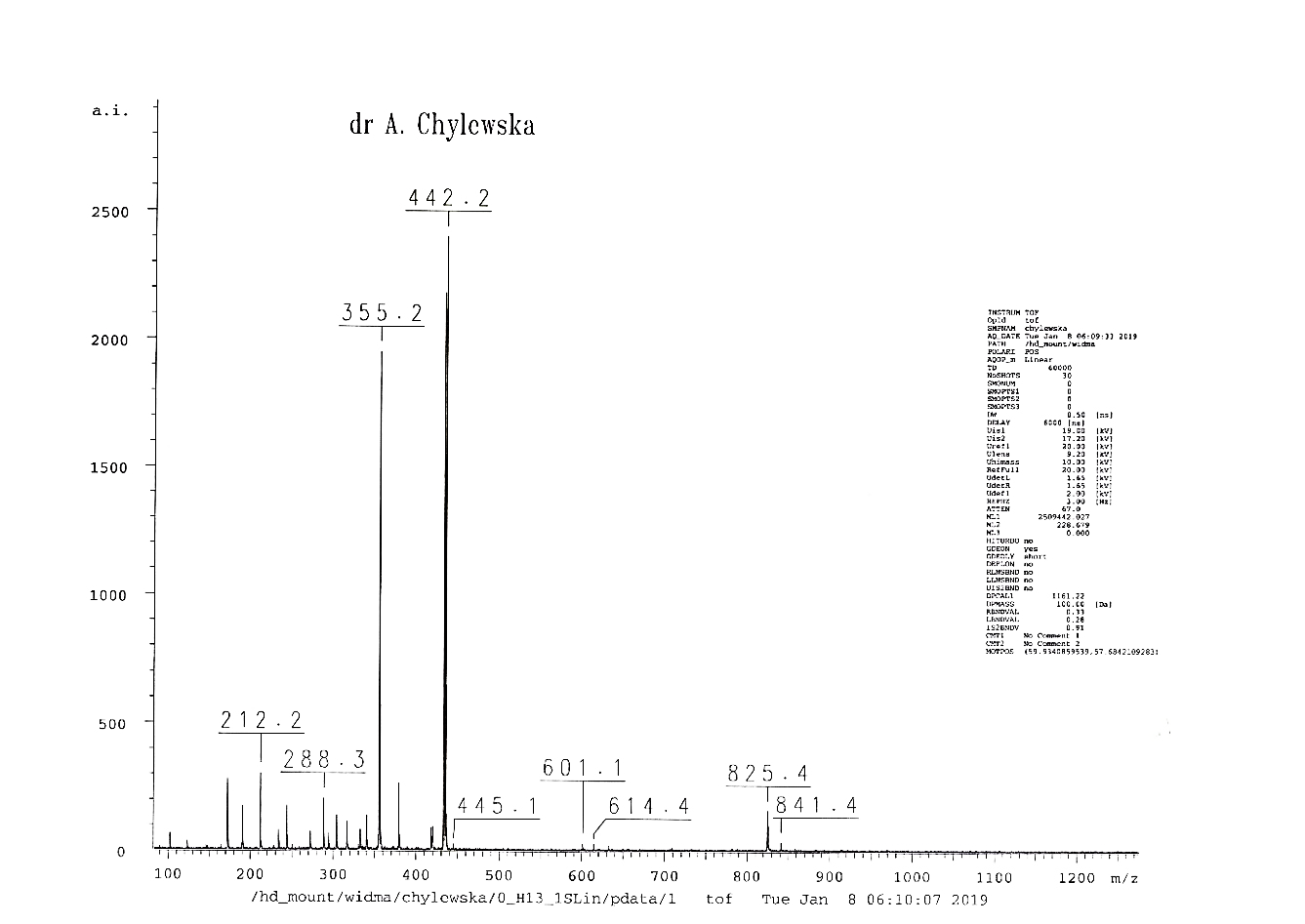


*Figure S21.* The MS/TOF spectrum of *3,6-PIRAMICAR* synthesized.

*Figure S22.* The ^1^H NMR spectrum of intermediate product **1**.

*Figure S23.* The ^1^H NMR spectrum of intermediate product **2** studied.

*Figure S24.* The ^1^H NMR spectrum of intermediate product **3** studied.


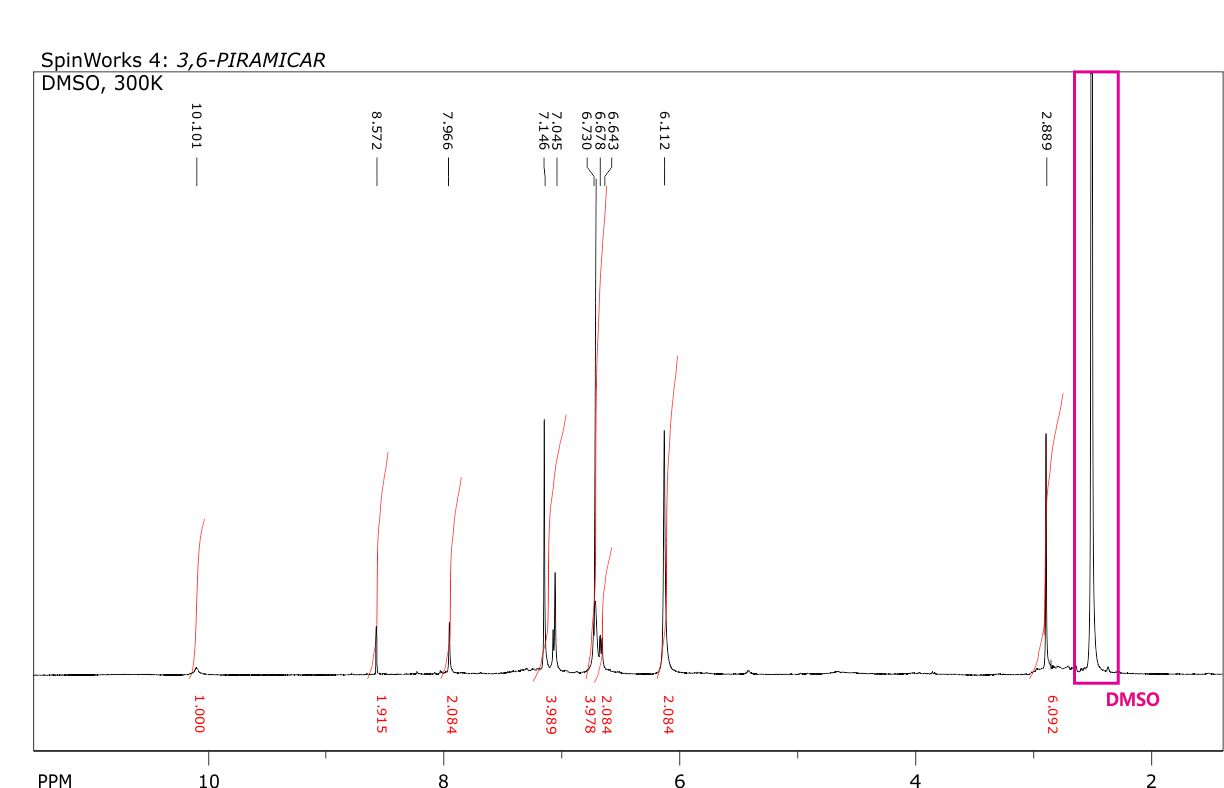


*Figure S25.* The ^1^H NMR spectrum of *3,6-PIRAMICAR* studied.


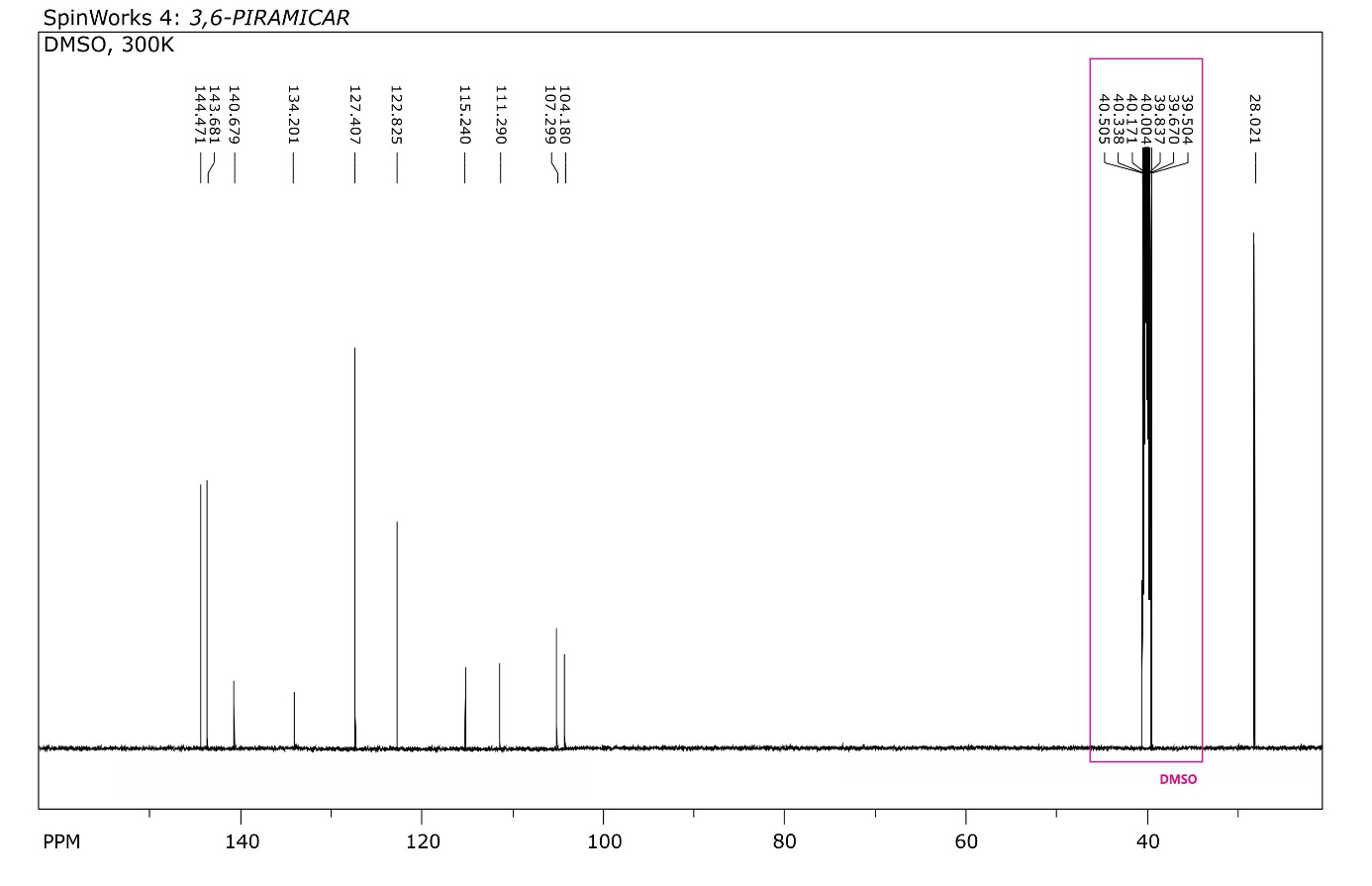


*Figure S26.* The ^13^C NMR spectrum of *3,6-PIRAMICAR* studied.


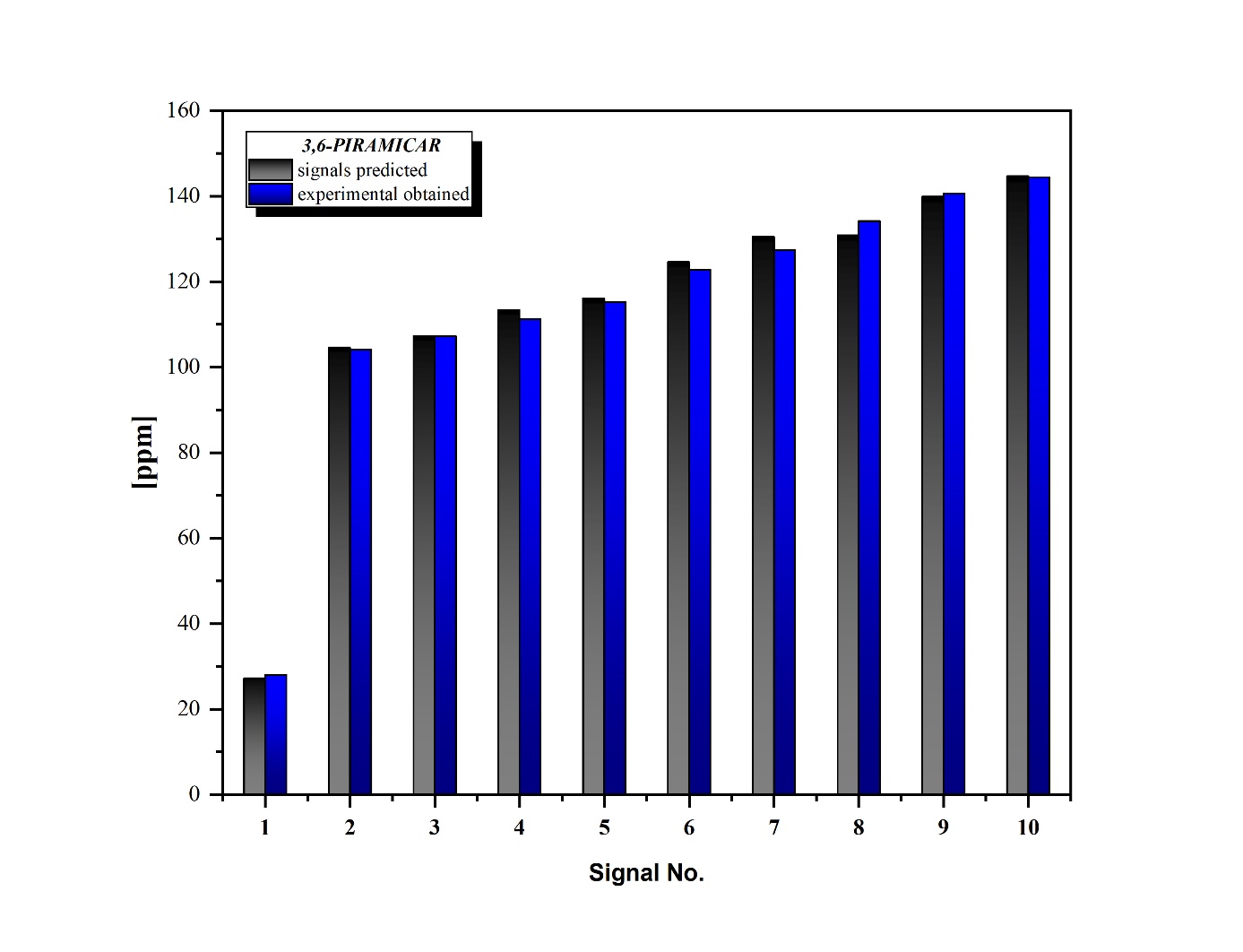
*Figure S27.* The comparison of ^13^C NMR spectra signals of *3,6-PIRAMICAR* studied.


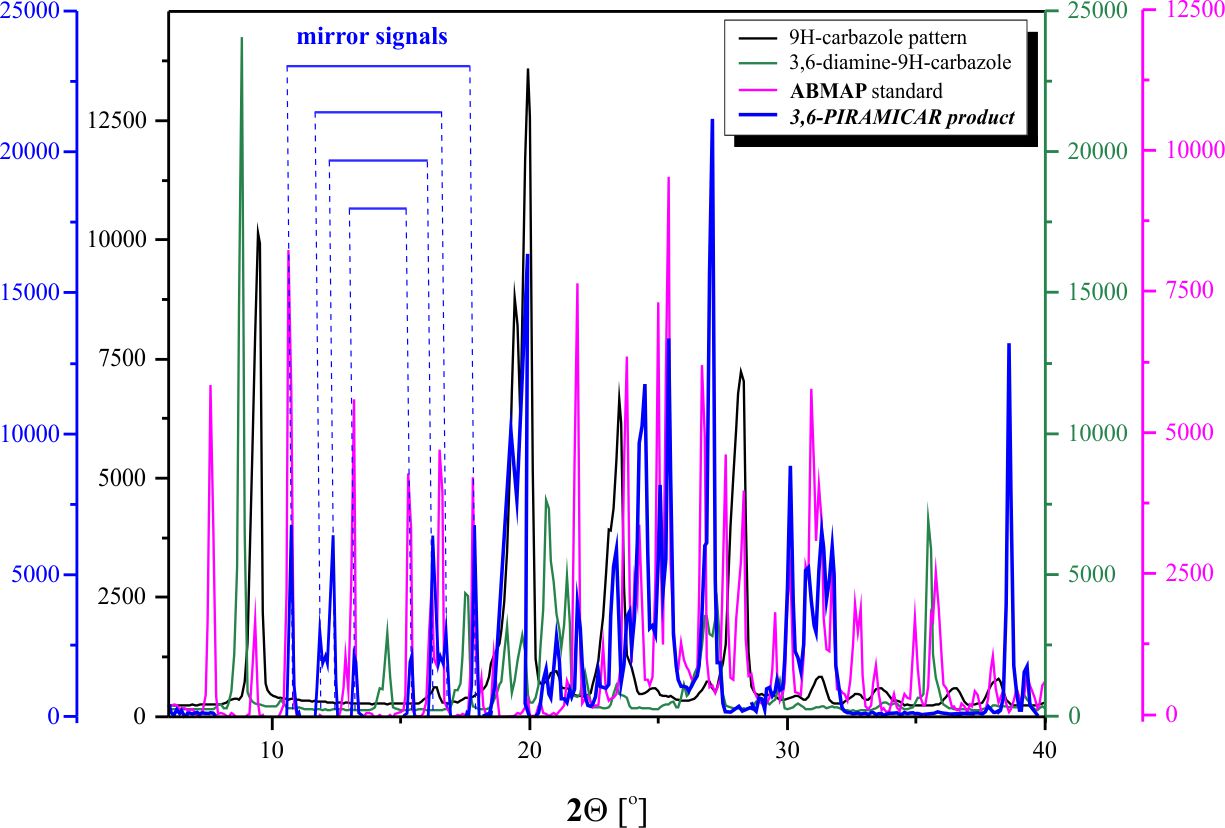


*Figure S28.* The XRD powder spectrum confirmed the symmetrical structure of *3,6-PIRAMICAR* studied.

***ATR vibrational analysis***

Although heteroaromatic compounds containing the amine group showed N-H stretching vibrations in the range 3500-3220 cm^-1^ like in the case of compound studied *3,6-PIRAMICAR*, the spectra of 9H-carbazole and 3,6-diamino-9H-carbazole show that the NH group presented in the carbazole ring vibrates and can be identified exactly at 3421 cm^-1^ (see *region A* marked in *Figure S29*). In the spectrum of *3,6-PIRAMICAR*, the bands at 3239 cm^-1^ and 3155 cm^-1^ were ascribed to NH_2_ group symmetry modes of vibrations, respectively [R5]. Moreover, the N-hetero aromatic compounds commonly exhibit C-H stretching vibrations in the region 3100-2900 cm^-1^ for asymmetric and symmetric stretching modes of vibration. In the present studies, the bands observed at 2938 cm^-1^ in the ATR spectrum was assigned for C-H symmetric stretching vibrations. The characteristic absorption of azomethine group ν(C=N) appeared at 1642-1566 cm^-1^ in the spectrum of *3,6-PIRAMICAR,* and band 1006 cm^-1^ could be attributed to C-N stretching vibrations [R6].


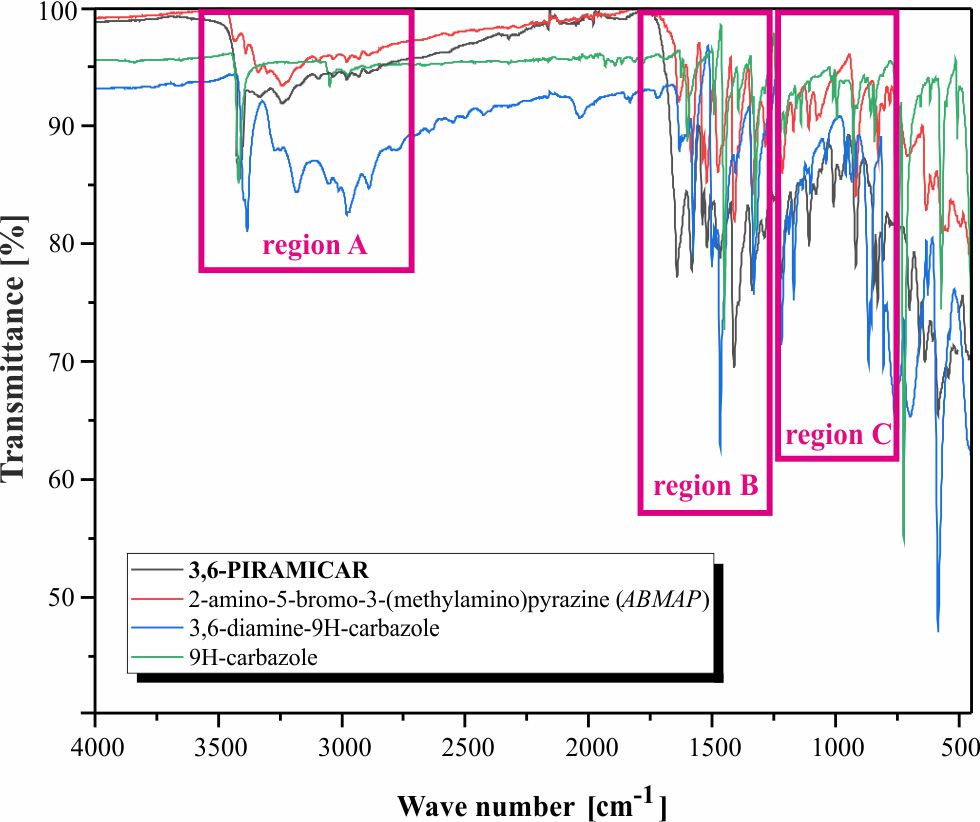


*Figure S29*. ATR spectra analysis of synthesized *3,6-PIRAMICAR* and its structural precursors.

The absorptions in the region of 1650-1400 cm^-1^ in the aromatic compounds were ascribed to carbon vibrations, see *region B* marked in *Figure S29* [R7]. The bands appeared at 1174 cm^-1^, and 1142 cm^-1^ in the spectrum of *3,6-PIRAMICAR*  could be attributed to the C-H in-plane bending vibrations [R8] and are related to pyrazine substituents (*region C* in *Figure S29*). The sharp bands at 921, 844, 825, and 811 cm^-1^ are related to the presence of C-H out-of-plane bending modes [R9]. Additionally, the characteristic absorptions of pyrazine ring C-C=N and C-N-C deformations were observed in the spectrum of *3,6-PIRAMICAR* at 779, 733, 700, 633, and 532 cm^-1^, respectively.

**References**

R1. Chylewska, A., Biedulska, M., Głębocka, A., Raczyńska, E. D., Makowski, M. Drug-like properties and complete physicochemical profile of pyrazine 2 amidoxime: A combined multi-experimental and computational studies. *J. Mol. Liq*. **276**, 453-470 (2019).

R2. Kostrowicki, J., Liwo, A. A general method for the determination of the stoichiometry of unknown species in multicomponent systems from physicochemical measurements. *Comp. Chem.* **11**, 195-210 (1987).

R3. Kostrowicki, J. Liwo, A. Determination of equilibrium parameters by minimization of an extended sum of squares. *Talanta* **37**, 645-650 (1990).

R4. Marquardt, D. W. An algorithm for least-squares estimation of nonlinear parameters. *J. Soc. Industrial Appl. Mathematics* **11**, 431-441 (1963).

R5. Silverstein, R. M., Morrill, T. C., Bassler, C. Spectrometric Identification of Organic Compounds. (John Wiley and Sons Ltd, 1991).

R6. Gunasekaran, S., Sailatha, E. Vibrational analysis of pyrazinamide. *Indian J. Pure Ap. Phy.* **47**, 259-264 (2009).

R7. Endrédi, H., Billes, F., Holly, S. Vibrational spectroscopic and quantum chemical study of the chlorine substitution of pyrazine. *J. Mol. Struct. THEOCHEM*  **633**, 73-82 (2003).

R8. Breda, S., Reva, I. D., Lapinski, L., Nowak, M. J., Fausto, R. Infrared spectra of pyrazine, pyrimidine and pyridazine in solid argon. *J. Mol. Struct.* **786**, 193-206 (2006).

R9. Yadav, U., Kumar, D. Study of heterocyclic compound – pyrrole. *IJRST* **5**, 68-73 (2015).
